# The effect of noninstrumental information on reward learning

**DOI:** 10.3758/s13421-024-01537-4

**Published:** 2024-02-23

**Authors:** Jake R. Embrey, Amy X. Li, Shi Xian Liew, Ben R. Newell

**Affiliations:** 1 School of Psychology, UNSW Sydney, Kensington, Australia; 2 Department of Experimental Psychology, University of Oxford, Oxford, UK; 3 School of Psychological Sciences, University of Melbourne, Melbourne, Australia

**Keywords:** Information seeking, Noninstrumental information, Information avoidance, Reward learning

## Abstract

Investigations of information-seeking often highlight people’s tendency to forgo financial reward in return for advance information about future outcomes. Most of these experiments use tasks in which reward contingencies are described to participants. The use of such descriptions leaves open the question of whether the opportunity to obtain such noninstrumental information influences people’s ability to learn and represent the underlying reward structure of an experimental environment. In two experiments, participants completed a two-armed bandit task with monetary incentives where reward contingencies were learned via trial-by-trial experience. We find, akin to description-based tasks, that participants are willing to forgo financial reward to receive information about a delayed, unchangeable outcome. Crucially, however, there is little evidence this willingness to pay for information is driven by an inaccurate representation of the reward structure: participants’ representations approximated the underlying reward structure regardless of the presence of advance noninstrumental information. The results extend previous conclusions regarding the intrinsic value of information to an experience-based domain and highlight challenges of probing participants’ memories for experienced rewards.

**Supplementary Information:**

The online version contains supplementary material available at 10.3758/s13421-024-01537-4.

## Introduction

Most of the time, the information we seek can directly inform our subsequent behaviour: finding a restaurant based on reviews, renting a new apartment based on location, or using Google Maps to find the fastest route home. Our desire for information, however, extends beyond these instrumental circumstances into domains where the information we seek has no obvious or immediate use. One such instance is our desire for information about the outcomes of unchangeable future events (Bennett et al., 2016; Charpentier et al., 2018; Iigaya et al., 2016; Kobayashi et al., 2019; Liew et al., 2022; Vasconcelos et al., 2015)—a type of *noninstrumental* *information*.

Seeking noninstrumental information is often costless; for example, checking election updates while the votes are still being tallied or the remaining time on a long-haul flight. Humans and nonhuman animals, however, are also willing to pay a price to obtain such information. For example, nonhuman animals prefer an information-rich option over a noninformative option, even when the former is less likely to yield reward (e.g., 20% vs. 50% probability; Stagner & Zentall, 2010; Vasconcelos et al., 2015; Zentall & Stagner, 2011; for review, see Dunn et al., 2023). Similarly, humans seek noninstrumental information about delayed rewards (Bennett et al., 2016; Iigaya et al., 2016; Liew et al., 2022) and punishments (Charpentier et al., 2018; Lanzetta & Driscoll, 1996; Liew et al., 2022; Zhu et al., 2017) and are willing to forgo financial reward (Bennett et al., 2016; Cabrero et al., 2019) or receive electric shocks (Lau et al., 2020) in order to obtain such information. In all of these instances, the information sought cannot reliably guide behaviour as the outcomes are predetermined; nevertheless, both people and animals are willing to pay a price to obtain it.

Theories of noninstrumental information seeking propose a range of factors which may underlie our affection for ‘useless’ information. For example, aversion to uncertainty (Bennett et al., 2016; Liew et al., 2022; van Lieshout et al., 2019), trait curiosity (Kobayashi et al., 2019; Loewenstein, 1994), and a desire to savour future rewards (Iigaya et al., 2016, 2020; Loewenstein, 1987). While these theories are distinct in terms of the mechanism posited to underlie information seeking behaviour, they share the assumption that information itself possesses intrinsic value (Grant et al., 1998; Kreps & Porteus, 1978); a commonality which separates them from standard theories of decision-making which ascribe value to information based on its ability to directly inform behaviour (Hirshleifer & Riley, 1979; Raiffa & Schlaifer, 1961).

**Fig. 1:**
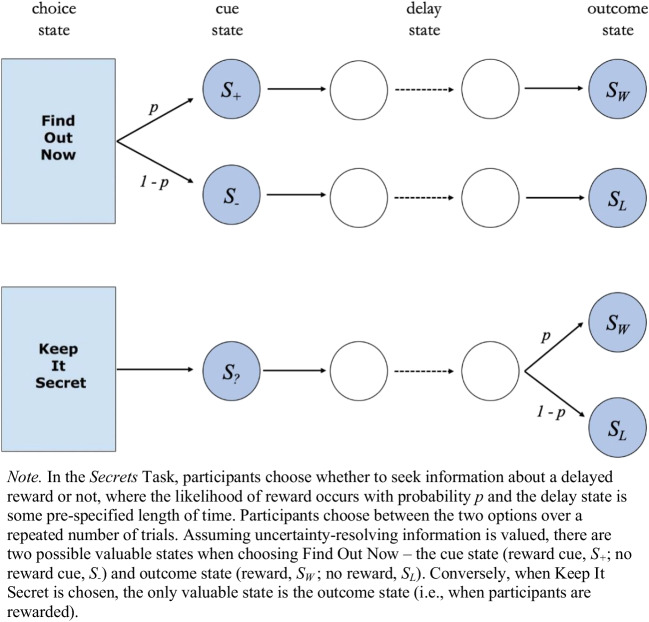
Schematic of the secrets task, commonly used to investigate noninstrumental information seeking

A task commonly used to illustrate the intrinsic value of information is the *secrets task*, which probes noninstrumental information preferences (Iigaya et al., 2016, 2020; Liew et al., 2022, 2023; Fig. 1). In this task, participants are given a choice between two options, Find Out Now (FON) and Keep It Secret (KIS). Choosing FON results in the immediate resolution of uncertainty about the delayed outcome, whereas choosing KIS provides no information about the delayed outcome. When all else is equal between the options (e.g., probability of outcome, delay duration), people typically show strong preferences for Find Out Now (e.g., Liew et al., 2022). Since the only difference between these options is the *cue states* (see Fig. 1)—which resolve outcome uncertainty for FON choices and maintain it for KIS choices—past work reasons that the information obtained, despite its noninstrumentality, must be valued.

Typically, experiments assessing people’s willingness to pay for noninstrumental information use explicit costs and rewards (i.e., participants agree to a specific amount they are willing to pay for information and the possible outcomes for each option are described). This stands in contrast to past studies conducted with nonhuman animals, in which the outcome contingencies of the options—and therefore the cost of seeking information—must necessarily be learned via sampling (the experimenter cannot tell a pigeon the outcome contingencies). Thus, although both human and animal work has found a willingness to pay for useless information—so called *suboptimal choice preferences—*a key distinction exists between the paradigms that give rise to these preferences. In human studies, the price paid for information is explicit and the possible outcomes of the two options are known; in animal studies, the outcomes of the options are learned via sampling, and it is unclear whether subjects are aware of the price they are paying to seek information.

Given that scenarios in which outcomes are learned from experience—rather than description—are ubiquitous in everyday settings, examining human choice preferences in paradigms more closely resembling those in the animal literature (i.e., outcomes learned via experience where one option is suboptimal) remains pertinent. Although this question has been addressed in some recent work (Molet et al., 2012; McDevitt et al., 2019; Stagner et al., 2020), these studies have yielded inconclusive results and used very small sample sizes (*n*s < 20).^1^ The current experiments aim to improve the methodology used to assess whether humans show suboptimal preferences when choice–outcome contingencies are learned, similar to the paradigms used in the animal literature.

In addition to behavioural evidence that humans and animals inherently value information, neuroimaging studies in humans and primates have found overlap in the neural structures which encode reward-relevant information prediction errors (IPEs) and reward prediction errors (RPEs)^2^ (see Bromberg-Martin & Monosov, 2020; van Lieshout et al., 2020, for an overview). For example, in primates, common midbrain dopamine neurons are associated with encoding both the IPEs from food-predictive cues and the RPEs upon food delivery (Bromberg-Martin & Hikosaka, 2009); and structures such as the lateral habenula have also been associated with both IPE and RPE encoding (Bromberg-Martin & Hikosaka, 2011). More recent work in humans using electroencephalogram (Brydevall et al., 2018) found both RPE and IPEs were reflected by the feedback-related negativity of event-related potentials, which indicates both IPEs and RPEs are encoded in a similar time-course in the brain.

**Fig. 2:**
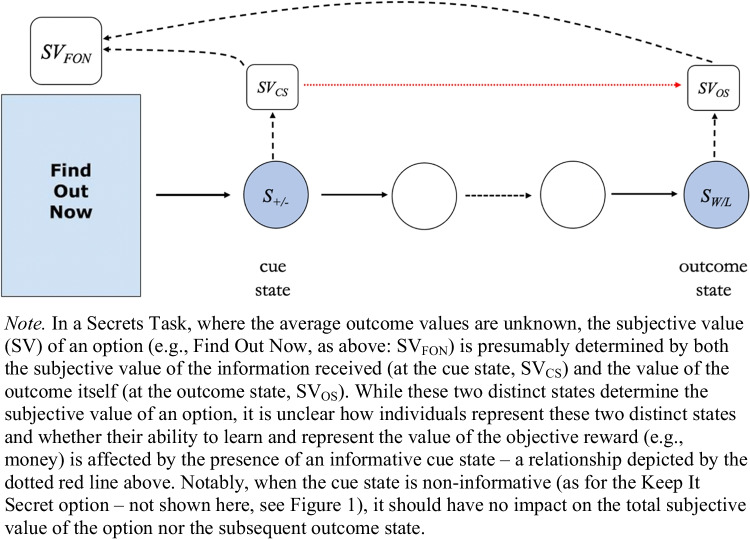
A schematic of how the subjective value of an option is determined in the secrets task

Given commonalities between neural architectures responsible for encoding reward and reward-relevant information, together with people’s tendency to overweight positive information (Kuzmanovic et al., 2016), it is plausible that people’s mental representations of how objectively rewarding an option is (e.g., the number of points received) are affected by the presence of intrinsically rewarding advance information. In other words, when learning the values of two options (e.g., Find Out Now and Keep It Secret) are people (and animals) able to distinguish the value imbued in an option (FON) by early information (at the cue state), and the value which is derived by the receipt of the delayed reward (outcome state)? Figure 2 shows a schematic describing how these states and values can be conceptualized within the secrets task.

Here, across two experiments, we investigated people’s propensity to seek information in a secrets task where participants learn the outcome contingencies of the options (Find Out Now and Keep It Secret) via experience, similar to the foraging-like tasks used in animal research (e.g., Vasconcelos et al., 2015). In addition to examining people’s preference for advance information, we were also interested in whether participants’ willingness to ‘pay’ for this information was driven purely by the intrinsically rewarding nature of information, or whether the presence of advance information affected participants’ ability to learn the objective reward (e.g., number of points) associated with an option. In the secrets task, participants need to be able to distinguish the values of payoffs (i.e., how much reward they receive from each option) from the value of the noninstrumental information available (i.e., the intrinsic value of eliminating uncertainty about a delayed outcome). Findings from standard decisions-from-experience paradigms suggest that participants’ representations of the reward values are often markedly different from the options’ true reward structures (Camilleri & Newell, 2013; Hertwig et al., 2004); the extent to which subjective representations of rewards align with objective payoff values is therefore an open question. Extending the secrets task paradigm thus enabled us to examine whether participants’ representations of option values were affected by the intrinsic value of any noninstrumental information associated with that given option.

## Experiment 1

Experiment 1 adapted the secrets task paradigm so the reward outcomes of both options were learned via sampling, rather than being described to participants in the instructions. Choosing the informative option (Find Out Now; FON), however, resulted in 33% less average reward than KIS (Keep it Secret; KIS); hence FON was suboptimal.

To contrast people’s choices in the task with choices in an analogous sampling task without the presence of advance information, we included a noninformative condition. Participants in this condition simply had a choice between two options (i.e., a square and a triangle) where the average reward for one option was 33% less than the alternative (i.e., it was suboptimal). Importantly, neither choice option offered the opportunity to resolve uncertainty about the upcoming outcome (hence the label ‘noninformative’).

**Fig. 3:**
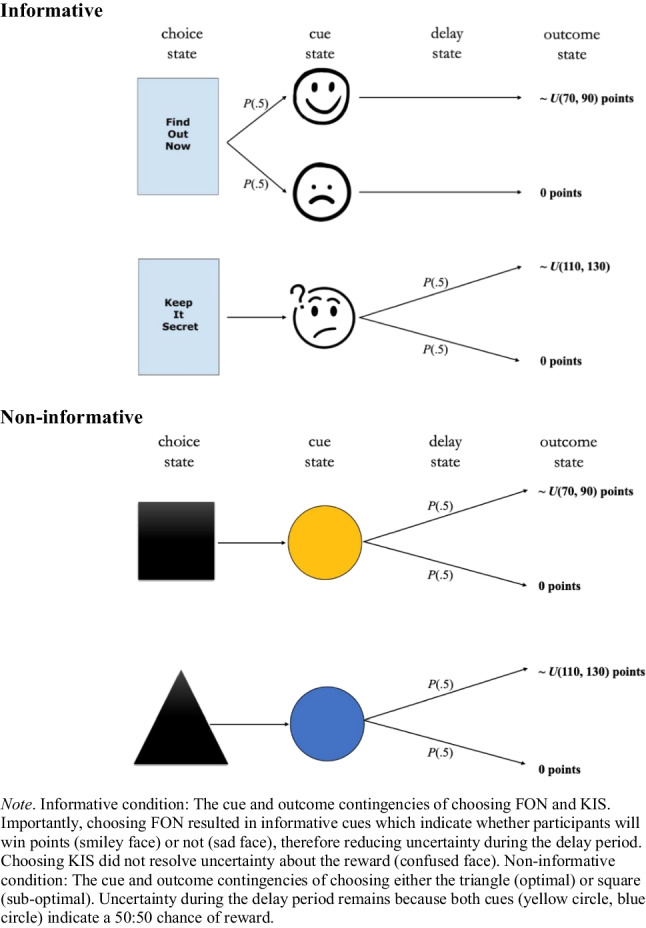
Schematic of the task structure in Experiment 1 for both conditions (**Informative**), (**Noninformative)**

Participants were provided with some information about reward contingencies (e.g., the 50-50 chance of reward or no-reward) and the informative nature of the cues—therefore learning was not exactly akin to the learning of animals in comparable tasks—but participants had to learn the choice–outcome contingencies (i.e., how many points an option was associated with) via sampling. We made this design choice because of our focus on the impact of noninstrumental information on the learning of outcome *value* rather than the *probability* of outcome receipt. See Fig. 3 for a schematic of the task design.

In light of past findings (Bennett et al., 2016; Cabrero et al., 2019; Charpentier et al., 2018), which find willingness to pay for information quickly diminishes as the cost increases, we did not anticipate a strong preference for FON (over KIS) in the informative condition as it required forgoing a substantial amount of potential earnings. We did however expect suboptimal choice preferences to be higher in the Informative condition compared with suboptimal preferences in the noninformative condition.

To determine whether increased preferences for FON were partially driven by misrepresentations of the objective reward value we probed participants’ beliefs about the outcome values (i.e., how many points they earned) for each option. In Experiment 1, this probe asked participants to estimate the average rewards they would receive when choosing Find Out Now and Keep It Secret, respectively. Participants in the noninformative condition were also required to give these estimates for the two options they faced (i.e., a square and a triangle stimulus).

Given the exploratory nature of the research question, we had no firm directional hypothesis as to whether participants’ estimates of the outcome values would differ between the informative and noninformative conditions. We therefore discuss two possible results below and the theoretical implications of each.

If participants’ reward estimates of the suboptimal option are higher when it is informative (FON) than when it is noninformative, this would suggest advance information interferes with people’s ability to learn the true reward structure of the environment. Participants may encode representative subjective values for an option (see SV_FON_ in Fig. 2) but not clearly distinguish between the two states which determine that option’s subjective value (both the cue and outcome state). Their representation of the objective reward (in this case, points) may be affected by the information received in the cue state.

Conversely, if participants’ estimates are equivalent across the two conditions, it would suggest that people’s abilities to learn the environment’s reward structure is unaffected by the presence of noninstrumental information. In this case, a willingness to forgo reward in order to seek information is simply a product of the value placed on advance information and not due to any obscuring effect information could have on people’s representations of the outcome values.

### Method

The experimental data and analysis scripts for both experiments (Experiments 1 and 2) can be found online (https://github.com/amyxli/nwtklearn).

#### Participants

A total of 101 adults participated in Experiment 1. Participants were recruited via Amazon Mechanical Turk (mTurk) and were paid $4.00 USD for their participation. In addition, participants were paid a bonus which depended on their earnings in the task (*M* = $3.02 USD). Participant assignment to each of the two conditions (informative, *n* = 49; noninformative,* n* = 52) in the experiment was randomized.

Participants were excluded if they failed the three-item attention check (which was conducted after the instructions; see Supplementary Materials for details) more than three times. This led to the exclusion of two participants. The effective sample size therefore consisted of 99 adults (37 female, 61 male, one other) with a mean age of 36.9 years (SD = 10.4, range: 23–73 years), with *n* = 47 in the informative condition, and *n* = 52 in the noninformative condition.

#### Materials

Experiments were conducted on participants’ own laptops or desktop computers (tablets or mobile devices were not permitted). The experiment was coded in jsPsych (De Leeuw, 2015) and JavaScript.

#### Design

The experiment was a between-subjects design consisting of two conditions: informative and noninformative. Both conditions contained four blocks of 15 trials for a total of 60 trials.

Participants in both conditions were required to choose between two options and learn the reward contingencies of the two options. In both conditions, both options resulted in ‘wins’ on 50% of trials and ‘no-wins’ (where zero points were awarded) on the remaining 50%. The average payoffs on the win trials, however, were not equal between the two options. For the suboptimal option, rewards on win trials were drawn randomly from a uniform distribution, ~*U*(70, 90) (a mean of 80 points). For the optimal option, rewards were drawn from a uniform distribution, ~*U*(110, 130) (a mean of 120 points). The average rewards for the two options, suboptimal and optimal, were therefore 40 points and 60 points, respectively (Fig. 3). We chose to use variable outcome values sampled from the aforementioned uniform distributions to ensure that learning choice–outcome associations would not be overly trivial; associating each option with a deterministic outcome value (e.g., suboptimal option ➔ 80 points and optimal option ➔ 120 points) could result in a ceiling effect in outcome value learning, such that the task might be insensitive to any effects of noninstrumental information.

**Fig. 4:**
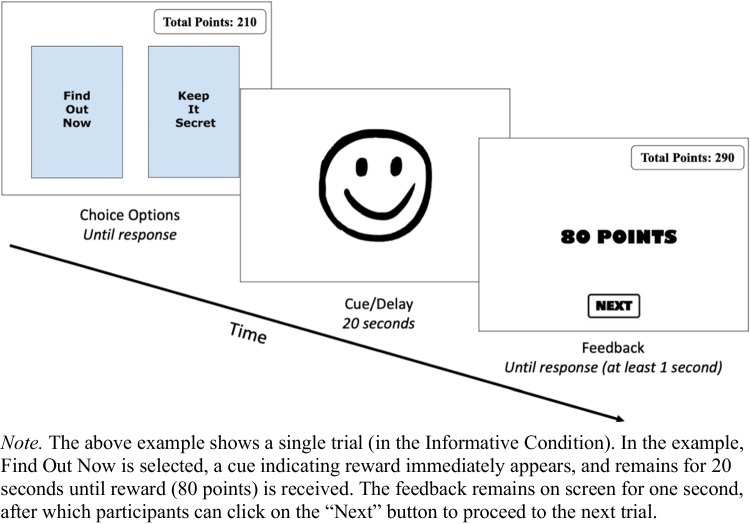
Example of a single trial in the choice phase

On every trial, the two choice options were available onscreen until participants made a selection. A cue was then presented for 20 seconds, followed by feedback about the outcome. This therefore resulted in a 20-second delay between choice and outcome for both conditions. Figure 4 shows an example of a single choice trial. The identity of the two options and the postchoice cues differed between informativeness conditions.

In the informative condition we used the secrets task paradigm (see Bennett et al., 2016; Iigaya et al., 2016) where, at the start of each trial, participants were required to choose between two options: Find Out Now (FON) or Keep It Secret (KIS). When choosing FON, participants immediately received a cue which informed them about the outcome (win or no-win) that would occur after the subsequent 20-s delay. Conversely, choosing KIS resulted in an ambiguous cue which provided no information as to the delayed outcome. These cues stayed on screen until point feedback was displayed. Point feedback was displayed for 1,000 ms for all trials.

Crucially, FON was always the suboptimal choice (average reward of 40 points) and KIS was always the optimal option (average reward of 60 points). Participants were not told these reward contingencies, but could learn them via sampling. They were however told that choosing FON led to informative cues (which predicted the outcome) and KIS kept the outcome unknown until the delivery of reward.

In the noninformative condition, participants were required to choose between two options: a square and a triangle. One of these options was always the suboptimal option, ~*U*(70, 90), mean 80, with the alternative being optimal, ~*U*(110, 130), mean 120. After choosing an option, a coloured cue (a blue or yellow circle) was displayed on screen and remained for the entirety of the delay period until the reward was displayed. Points feedback was displayed for 1 000 ms for all trials. The colour of the cue depended on which option was chosen (e.g., square ➔ blue circle, triangle ➔ yellow circle), but the cue gave no information as to what the delayed outcome would be. The mapping between shape and cue colour, as well as the mapping between shape and relative option optimality, were randomized between participants.

Following the main task, outcome estimates were used to probe participants’ representations of the rewards associated with the two options. Participants were shown each of the two options they learned about in the task (e.g., FON for a participant in the informative condition) and were asked “What do you estimate the *average win* to be when you chose the above option (excluding 0-point outcomes)?”. Estimates were given as an integer and were unbounded—participants could input any value greater than or equal to zero.

#### Procedure

All participants were instructed that they would complete a decision-making task consisting of two options. Participants were informed that the choices would result in point rewards which were equivalent to actual money which would be awarded to them upon completion—1,000 points = $1.00 USD.

In the informative condition, participants were told these two options and the predictive nature of the FON option. Specifically, they were told choosing FON resulted in either a happy or sad face, which indicated whether they would win points or receive 0 points after the 20-second delay. They were also told that choosing KIS resulted in a confused face which provided no information as to whether points would be won or not. Participants were however provided with no information as to the average rewards of the two options and whether their choices (FON or KIS) impacted their earnings.

In the noninformative condition, participants were told they would choose between two options, a square and a triangle. They were also told they would receive a coloured cue after their choice which corresponded to their choice. They were provided with no information as to the average rewards of the two options and whether their choices determined their earnings in the task.

Participants in both conditions were told that both options resulted in wins on 50% of trials and no-wins on the remaining 50%. We explicitly informed participants about this stochastic design feature to reduce a potentially confounding source of response variability: participants’ belief that there is a pattern to the choice–outcome payoffs, which has been found to arise in decisions-from-experience tasks (see Szollosi et al., 2023).

After the instructions, participants completed a three-item attention check (see Supplementary Materials for full details). Participants were required to get all three questions correct prior to moving to the main task. Participants were not told they would be asked about the value of the outcomes received during the task.

Participants then underwent the choice trial phase which consisted of 60 choices (four blocks of 15) between the two options. During this phase, the total amount of points earned were displayed in the top-right of the screen (Total Points: *N* points).

After completing the 60 choice trials, participants provided the outcome estimates for the two options in their task. In both conditions, participants gave separate value estimates for each of the two options they learned about during the task (i.e., FON and KIS for the informative condition; a square and triangle for the noninformative condition). For each option, they were asked to estimate the average amount of points they won on ‘win-trials’ for that option (see Design for details).

Upon completion, participants were debriefed and were awarded their bonus payments, which depended on their choices in the task.

#### Data analysis

All data were analyzed using the statistical computing software R (R Development Core Team, 2022). Generalized linear mixed models were fitted using the lme4 package (Bates et al., 2015), likelihood ratio tests conducted using the stats package (R Development Core Team, 2022), and two-way analyses of variance (ANOVAs) were conducted using the rstatix package (Version 0.7.2; Kassambara, 2023).

### Results

#### Preference for suboptimal choices

**Fig. 5:**
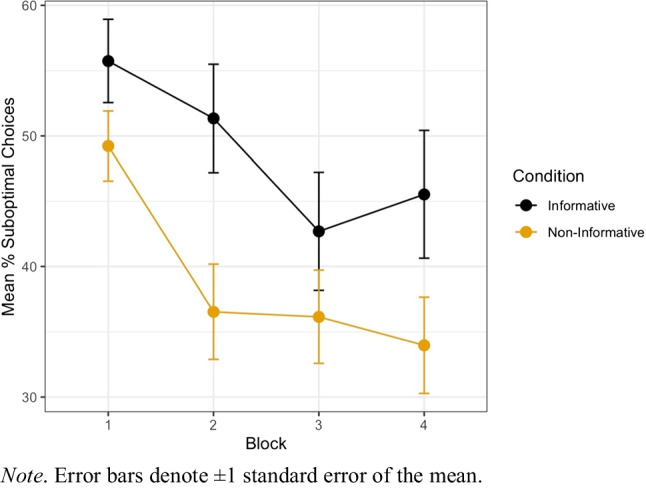
Participants’ mean percentage of suboptimal choices across blocks in Experiment 1

First, we examined whether participants’ preferences for the suboptimal option differed between the informative and noninformative condition. Figure 5 shows the mean percentage of suboptimal choices between the two conditions.

Participants’ choices on each trial were analyzed using a generalized linear mixed model. In this model choice was treated as the binary outcome variable (suboptimal or optimal), with condition (noninformative vs. informative) and trial number as fixed effects, and random intercepts for participants. This model indicated a significant main effect of condition, such that those in the Informative condition were significantly more likely to choose the suboptimal option than those in the noninformative condition, *OR* = exp($\beta$) = 1.76, 95% CI [1.03, 2.98], *z* = 2.08, *p* = .037. In addition, a significant main effect of trial was found, indicating that participants’ propensity to choose the suboptimal option declined as the task progressed, *OR* = exp($\beta$) = 0.985, *z* = −8.950, *p* < .001. A more complex model incorporating an interaction term between condition and trial was not found to be significantly different to the simpler model above, χ^2^(1) = 0.1971, *p* = .657.

#### Analysis of win-stay, lose-shift behaviour

Given the nature of the task, it is possible participants’ behaviour followed a win-stay, lose-shift policy. We analyzed the relationship between participants’ previous outcome and subsequent choice using a generalized logistic regression. To do this, for all trials excluding the first (for which there was no previous choice), we calculated whether a participant stuck with their previous choice (labelled ‘0’) or switched (labelled ‘1’). The generalized logistic regression was of the form *switch ~ previous reward × condition* + (1 | *subject*), to assess the relationship between participants’ previously experienced reward and their propensity to switch or stay.

This analysis revealed a significant effect of *previous reward* such that participants were more likely to stay with their previous choice the larger the previous reward received was: *OR* = exp $(\beta )$ = .995, *z* = −5.10, *p* < .001. There was, however, no main effect of *condition* (indicating stay-switch behaviour did not differ between groups) and no interaction between *condition* and *previous reward.*

We also calculated the *P*(stay | win) and *P*(switch | lose) values for each participant. These are calculated across all trials by calculating the proportion of times they stayed with their previous choice when the reward was greater than zero points, and the proportion of times they switched from their previous choice when they received no reward (0 points). For the informative condition: *P*(stay | win) was .69 (*SD* = .23) and *P*(switch |lose) was .38 (*SD* = .20). For the noninformative condition: *P*(stay | win) was .68 (*SD* = .21) and *P*(switch | lose) was .40 (*SD* = .22).

#### Post-test value estimates

Next, we examined participants’ post-test estimates of the mean outcomes (for win trials) associated with both the suboptimal and optimal options. Recall that the average reward on win trials was 80 points ~ *U*(70, 90) for the suboptimal option, and 120 points ~ *U*(110, 130) for the optimal option.

**Fig. 6:**
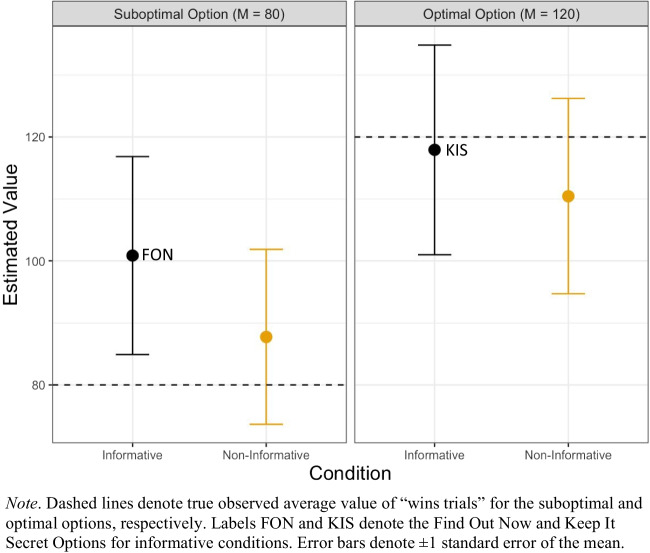
Participants’ post-test estimates of mean observed outcome values of “win trials” in Experiment 1

To exclude improbable estimates, participants who gave points estimates of 0 (which they were explicitly asked not to do) or more than 1,000 (the maximum possible reward earned on any one trial was 130) were excluded from both visualization and analyses. This led to the exclusion of 19 participants and an effective sample size of 80. Average estimates from participants following exclusion are shown in Fig. 6.

We then ran a two-way mixed ANOVA on participant estimates, with option type (optimal vs. suboptimal) as a within-subjects factor and informativeness condition (informative vs. noninformative) as a between-subjects factor. Participant estimates were log-transformed prior to the ANOVA.

We did not find evidence for a significant difference in post-test estimates between the informative versus noninformative conditions, *F*(1, 78) = 0.46, *p* = .498, η^2^ = .005. There was a significant main effect of option type, *F*(1, 78) = 10.29, *p* = .002, η^2^ = .022, reflecting higher estimates for the optimal option than for the suboptimal option. There was no significant interaction effect between informativeness condition and option type, *F*(1, 78) = 0.404, *p* = .527, η^2^ = .0009.

#### Relationship between post-test estimates and choice

Additionally, we analyzed the relationship between participants’ self-report estimates and their choice preferences in the task. Difference scores were obtained for each participant: these were calculated by taking each participant’s estimate for the suboptimal option and subtracting their estimate for the optimal option (i.e., *difference* = *estimate*_*suboptimal*_* − estimate*_*optimal*_). A linear regression was then fit predicting participants’ proportion of suboptimal choices (across the experiment) by their difference score: *P(suboptimal) ~* 1 + *difference*. We expected a positive relationship as the more rewarding an individual believed the suboptimal option to be, relative to the optimal option, the more they should choose suboptimally.

We observed a weak, nonsignificant positive relationship between the difference score and participants’ choices in the task, *β =* 0.075, *t*(78) = 1.968, *p* = .053.

### Discussion

The results of Experiment 1 show that people are willing to forgo reward to seek advance information when the outcome contingencies are learned, rather than explicit as in previous work (e.g., Bennett et al., 2016). Participants in Experiment 1 chose the suboptimal option (which had a mean value 33% lower than the optimal option) significantly more when it resulted in advance information compared with when the option was noninformative. Given this pattern of choice preferences, the subjective value of the suboptimal option is presumably higher when it is informative (FON) as opposed to when it is not.

The results of Experiment 1 found no evidence in favour of the hypothesis that participants’ mental representations of the outcomes were affected by the presence of advance information. We observed no difference between the informative and noninformative condition in terms of the outcome estimates. Participants’ estimates were however associated with their preferences in the task (albeit weakly), suggesting that their beliefs about how rewarding an option is (in terms of the points won) was related to their likelihood of choosing that option.

We note that only one single-value estimate was obtained (after the task’s completion) for each of the two observed options, and that these estimates were noisy—even after participants who gave extreme estimates were removed. It is therefore difficult, on the basis of this experiment, to confidently conclude that advance information has no effect on people’s representations of an option’s value.

In Experiment 2, we expanded the methods used to obtain outcome estimates as well as obtaining estimates both during and after the choice task. Additionally, we changed to an undergraduate student population as opposed to collecting data from an online mTurk population (where the quality of participant data has been questioned^3^).

## Experiment 2

The aims of Experiment 2 were the same as Experiment 1: to assess whether the presence of early information influences people’s ability to learn the underlying reward structure of an environment. Specifically, Experiment 2 aimed to improve the methods used to probe participants’ underlying value representations of the options in the task.

First, we added a probe during the choice phase of the experiment. In Block 4 (i.e., the last 15 choices), participants were asked to estimate how many points they would win by choosing each option, assuming it was a ‘win trial’. Additionally, upon the completion of the choice phase, participants were required to make repeated estimates for each option, for both ‘win’ and ‘no-win’ trials, as to how many points they expected to receive. We intended these questions to give an indication as to how often participants expected ‘win trials’ and ‘no-win trials’, as well as how much they expected to win when they did. See Methods for further details on these two procedures.

We also manipulated the amount of reward associated with each option. Rather than the informative option (FON) always being suboptimal, we included conditions where it was worth an equal amount, or worth more than the alternative (KIS). These changes allowed us to examine how the underlying reward structure affects preferences for noninstrumental information, in addition to our aim of assessing how the presence of noninstrumental information influences representations of the reward structure.

**Fig. 7:**
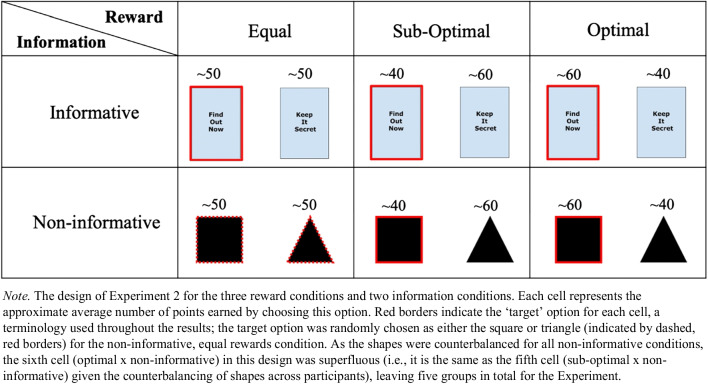
Design of Experiment 2

Experiment 2 was a 2 × 3 between-subjects design, with two levels of informativeness (either a noninformative or informative condition) and three levels for the reward environment (suboptimal, equal rewards, and optimal). Only five conditions were run, however, since the ‘sixth cell’ was superfluous. See Fig. 7 and the Methods for further details.

Following Experiment 1, we anticipated increased choices of FON, regardless of its value, compared with its noninformative counterpart. Following the results of Experiment 1’s outcome estimates, we did not expect any systematic difference between participants’ estimates as a function of the option’s informativeness.

### Method

#### Participants

A total of 255 people participated in Experiment 2. Participants were first-year psychology students at UNSW who were compensated for their participation via course credit. In addition, participants were paid a bonus which depended on their earnings in the task (*M* = $3.03 AUD).

Following Experiment 1, participants were excluded if they failed the three-item attention check more than three times; two participants were excluded from Experiment 2. The effective sample size therefore consisted of 253 adults (164 female, 83 male, eight other) with a mean age of 19.92 years (*SD* = 3.58, range: 17–40 years).

#### Materials

The experiment was coded using jsPsych (De Leeuw, 2015). Participants completed the task via a web browser on their own laptop or desktop computers. Mobile devices were not allowed.

#### Design and procedure

The experiment consisted of five conditions (see Fig. 7), which were run between subjects, to assess how the presence of advance noninstrumental information affected participants’ choice preferences across differing reward structures.

The design and procedure of the task was the same as Experiment 1, with exceptions noted here. The choice phase consisted of four blocks, each of which had 15 trials where participants could freely choose either option. In the fourth block, participants were asked on each trial to estimate how many points they believed they would win, for both options, if it was a ‘win trial’ (i.e., where the participant won greater than 0 points).

After the choice phase we further probed participants’ representations of the reward structure by having them give repeated estimates (10 in total) of the outcomes for both options in the task (i.e., FON and KIS for those in informative conditions and the square and triangle for those in the noninformative conditions). Here, participants were asked to give responses for both ‘win’ and ‘no-win’ trials.

Participants were randomly assigned to one of the five conditions: informative, target suboptimal (*n* = 50); informative, target optimal (*n* = 50); informative, target and nontarget equal (*n* = 49); noninformative, target and nontarget unequal (*n* = 50); noninformative, target and nontarget equal (*n* = 54). Note that the noninformative, unequal condition is used as the noninformative comparison group for both the informative, target suboptimal and informative, target-optimal conditions (see Fig. 7).

### Results

We use the terms ‘target’ and ‘nontarget’ throughout the Results section. For all informative conditions, the ‘target’ is Find Out Now and the ‘nontarget’ is Keep It Secret. For the noninformative conditions, the ‘target’ changes depending on the reward structure. For the equal reward condition, the target is randomly allocated as either the square or triangle (because each is associated with the same reward: 50 points). For the noninformative unequal rewards conditions, the target is the suboptimal option (40 points) when comparing to the suboptimal, informative condition, and is the optimal option (60 points) when comparing to the optimal, informative condition. See Fig. 7 for details.

#### Choice preference

**Fig. 8:**
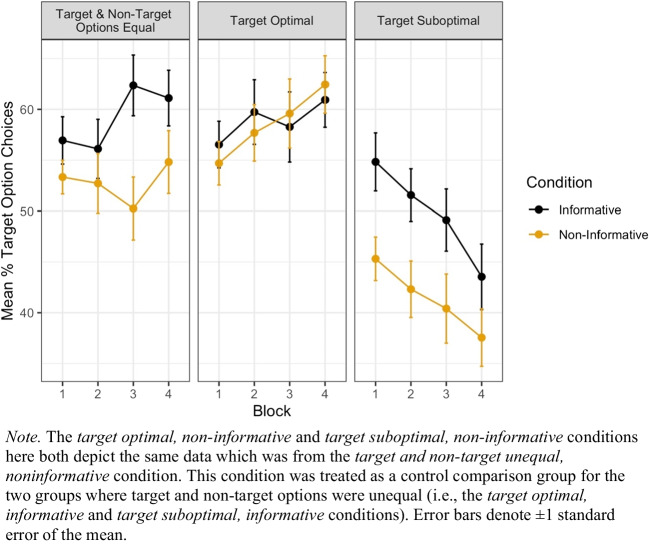
Participants’ mean percentage of target choices across blocks in Experiment 2

First, we examined whether choice preferences were influenced by the presence of advance information. Figure 8 shows the mean percentage of trials on which participants chose the target option for all conditions.

Participants’ choices on each trial were analyzed using a generalized linear mixed model separately for each of the optimality conditions. In each model, choice was treated as the binary outcome variable (choosing the target vs. nontarget option), the informativeness condition (noninformative vs. informative) and trial number were fixed effects, and participant was included as a random effect. As with the analysis of choice data from Experiment 1, for each model, we used a likelihood ratio test to compare the fit of the main-effects-only model against a model with an additional interaction term between trial number and informativeness condition.

When the target (i.e., FON for the *informative* condition, and either square or triangle for the *noninformative* condition) and nontarget (KIS for the *informative* condition) were equal in average rewards, the informativeness of the cues, *OR* = exp($\beta )$ = 1.32, 95% CI [1, 1.74], *z* = 1.97, *p* = .0491, was a significant predictor of choice with those in the informative condition choosing the target more than those in the noninformative condition (Fig. 8, leftmost panel). Trial number was also a significant predictor of choice with choices for the optimal option increasing across trials, *OR* = exp($\beta )$ = 1.003, *z* = 2.07, *p* = .0385. A more complex model incorporating an interaction term between condition and trial was not found to be significantly different to the simpler model above, χ^2^(1) = 2.157, *p* = .142).^4^

When the target option was associated with suboptimal outcomes, the informativeness of the cues, *OR* = exp($\beta )$ = 1.46, 95% CI [1.12, 1.91], *z* = 2.803, *p* = .005, was a significant predictor of choice. Additionally, trial number was also a significant predictor of choice, *OR* = exp($\beta$) = 0.991, *z* = −5.79, *p* < .001, with choice for the suboptimal option decreasing over trials. A model incorporating an additional interaction between condition and trial did not improve model fit relative to the simpler model above, χ^2^(1) = 0.387, *p* =.534. This replicates the patterns found in Experiment 1 (compare Fig. 8 rightmost panel with Fig. 5).

When the target option was associated with optimal outcomes, the informativeness of the cues was not a significant predictor of choice, *OR* = exp($\beta$) = 1.01, *z* = 0.053, *p* = .957. Trial number, however, was a significant predictor of choice, *OR* = exp($\beta$) = 1.005, *z* = 3.61, *p* < .001, such that participants were more likely to choose the target option (i.e., optimal) with each subsequent trial (Fig. 8, middle panel). A model incorporating an additional interaction between condition and trial did not improve model fit relative to the simpler model above, χ^2^(1) = 2.292, *p* = .130.

#### Analysis of win-stay, lose-shift behaviour

Following Experiment 1 we analyzed how participants’ current choice was affected by the reward received on their previous choice. We ran a generalized logistic regression model of the form, *switch ~ previous reward × condition* + (1 | *subject*), where condition was either informative or noninformative. We collapsed across optimality conditions (where the target was optimal, suboptimal, or equal in reward) for this analysis.

The analysis showed a significant effect of *previous reward* where participants were more likely to stay with the previous choice the higher the reward received on the previous trial, *OR* = exp($\beta )$ = .9983, *z* = −3.87, *p ≤* .001. There was however no population level effect of *condition* (either informative or noninformative) and no interaction between the two terms.

We also calculated the parameter values—*P*(stay | win) and *P*(switch | lose)—for the informative and noninformative conditions collapsed across optimality. For the informative condition: *P*(stay | win) was .58 (*SD* = .17) and *P*(switch | lose) was .45 (*SD* = .15). For the noninformative condition: *P*(stay | win) was .58 (*SD* = .18) and *P*(switch | lose) was .46 (*SD* = .17).

#### In-task value estimates

**Fig. 9:**
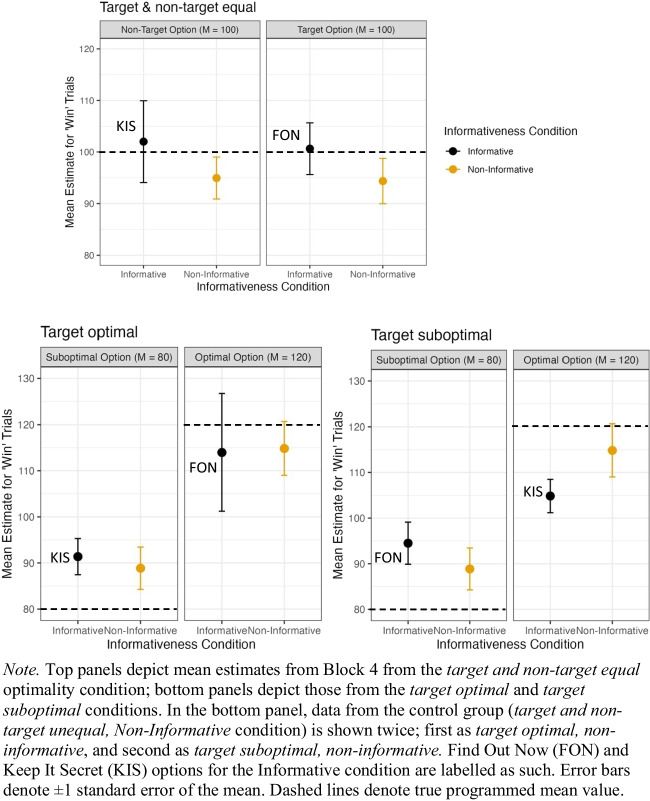
Participants’ in-task estimates of outcome values for “win trials” during Block 4 in Experiment 2

To exclude improbable estimates provided during Block 4, we implemented two exclusion criteria for visualization and analyses. First, estimates greater than or equal to 1,000 were excluded from visualization and analyses (as in Experiment 1). This led to the exclusion of 10.67% of the data. Secondly, as participants were asked to provide estimates for ‘win’ trials only, estimates of zero were interpreted as participants misunderstanding the instructions and erroneously providing estimates for ‘no-win’ trials; thus, these estimates were also excluded, leading to the exclusion of a further 7.43% of the data. In total, 18.10% of the data were excluded. Estimates from participants following the two exclusion criteria are shown in Fig. 9.

We analyzed participants’ outcome estimates in Block 4 using a linear mixed model, with each individual value estimate as the dependent variable. For each optimality condition, a separate linear mixed model was run with option type (target vs. nontarget), informativeness condition, and their interaction term as fixed effects, and with random intercepts for subjects.

First, we examined the case where the target and nontarget were equal in average outcomes (Fig. 9, top panel). We did not find a significant effect of either option type (*p* = .858), informativeness condition (*p* = .451), nor their interaction term (*p* = .761).

Second, we examined the case where the target option was associated with optimal outcomes compared with the nontarget option (Fig. 9, lower left panel). We found a main effect of option type, *β* = 25.221, *t*(2280.417) = 7.648, *p* < .001, such that the target option (the optimal option) received higher estimates. There was no main effect of informativeness condition, *β* = 7.848, *t*(56.664) = 0.822, *p* = .415. A significant interaction between option type and informativeness condition was found, *β* = −12.964, *t*(2270.207) = −2. 853, *p* = .004, because estimates in the informative condition were higher than in the noninformative condition for the suboptimal option, but not for the optimal option.

Third, we examined the case when the target option was associated with suboptimal outcomes compared with the nontarget option (Fig. 9, lower right panel). We found a main effect of option type, *β* = −25.08, *t*(2336.106) = −6.643, *p* < .001, such that the target option (i.e., the suboptimal option) received lower estimates than the nontarget. However, there was no main effect of informativeness condition, *β* = −9.235, *t*(−1.464) = −0.190, *p* = .145. We found an interaction between informativeness condition and option type, *β* = 11.388, *t*(2331.637) = 2.154, *p* = .031, such that estimates in the informative condition were higher than in the noninformative condition for the suboptimal option, but the reverse was true for the optimal option. That is, estimates were closer to the true observed mean values in the noninformative condition than the informative condition.

#### Post-test repeated value estimates

**Fig. 10:**
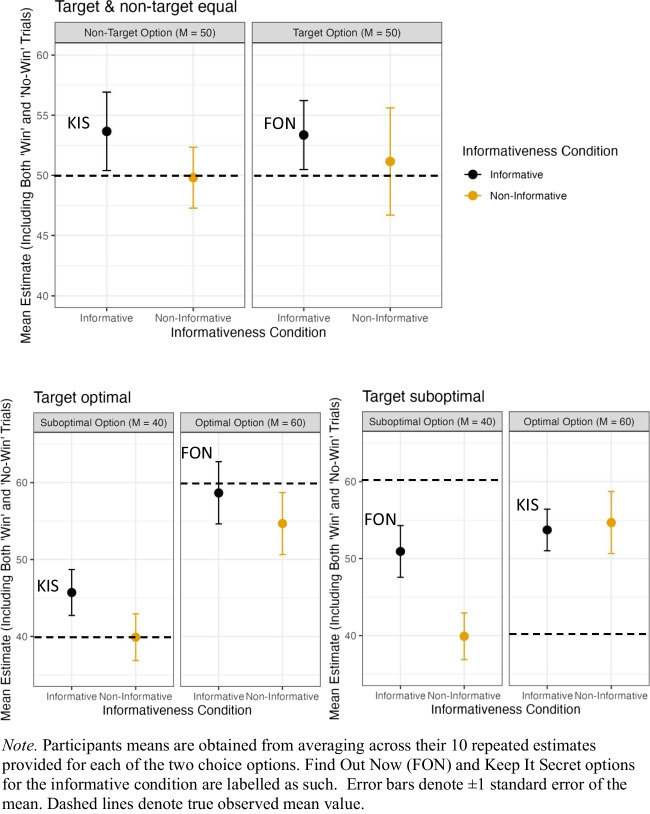
Participants’ mean repeated estimates of the mean observed outcome values across both ‘win trials’ and ‘no-win trials’ in Experiment 2

Participants were also asked to make 10 repeated estimates (including both ‘win’ and ‘no-win’ outcomes) of each option in the experiment after the choice phase (see Methods for details). Improbable estimates of greater than or equal to 1,000 were excluded, resulting in the removal of 0.17% of total data. Those who failed to provide at least one zero-value estimate for each of the two response options (*n* = 91) were also excluded, leading to an effective sample size of 164. Estimates from participants following exclusion are shown in Fig. 10.

We analyzed participants’ outcome estimates using the same model as we used for the Block 4 estimates (described above).

First, we examined the case where the target and nontarget were equal in average outcomes (Fig. 10, top panel). We did not find a significant effect of either option type (*p* = .950), informativeness condition (*p* = .416), nor their interaction term (*p* = .792).

Second, we examined the case where the target option was associated with optimal outcomes compared with the nontarget option (Fig. 10, lower left panel). We found a main effect of option type, *β* = 12.942, *t*(1159) = 3.005, *p* = .003, such that the optimal option received higher estimates than the suboptimal option. However, no effect of informativeness condition, *β* = −5.809, *t*(143.922) = −1.126, *p* = .262, nor the interaction term, *β* = 1.828, *t*(1159) = 0.298, *p* = .766, were found.

Third, we examined the case when the target option was associated with suboptimal outcomes compared with the nontarget option (Fig. 10, lower right panel). We did not find a main effect of informativeness condition, *β* = 0.957, *t*(190.502) = 0.203, *p* = .840, nor option type, *β* = −2.794, *t*(1254) = −0.678, *p* = .498. There was a marginally significant effect of the interaction between informativeness condition and option type, *β* = −11.98, *t*(1254) = −1.959, *p* = .050, such that estimates were higher for the informative condition than the noninformative condition, but only for the suboptimal option.

Analogous to Experiment 1 we also obtained single-point estimates for win-trials for all conditions. This analysis leads to similar conclusions to the above (i.e., no systematic or consistent bias towards overestimating the value of informative options, Find Out Now, compared with noninformative equivalents). The details of this analysis are in the Supplementary Materials.

### Relationship between outcome estimates and choice

Following Experiment 1, we assessed the relationship between people’s estimates and their choice preferences in the task. Below we report analyses for both the in-task (Block 4) and post-task repeated estimates.

To assess the relationship between participants’ in-task estimates and their choice preferences we calculated the mean estimate for each participant for both the target and nontarget option. We subtracted their mean estimate for the nontarget from their mean estimate for the target to attain difference scores. A linear regression was carried out assessing their proportion of target choices across the experiments, *P(target) ~* 1* + difference,* which found a significant positive relationship between the difference in participants’ average Block 4 estimates and their average preference for the target option in the experiment, *β* = 0.146, *R*^*2*^ = 0.077, *F*(1, 249) = 20.63, *p* < .001.

Similarly, we assessed the relationship between participants’ post-task estimates and their preferences in the choice phase. For the post-task repeated estimates we averaged their guesses (across both zero and nonzero estimates) for both the target and nontarget option. We then calculated a difference score for each participant by subtracting their mean estimate for the nontarget by their mean estimate for the target. A linear regression, *P(target) ~* 1* + difference*, found a significant positive relationship, *β* = 0.219, *R*^*2*^ = 0.098, *F*(1, 160) = 17.48, *p* < .001, between participants’ difference scores and their propensity to choose the target option. Additional figures and statistics are provided for both of the above in the Supplementary Materials.

### Discussion

Experiment 2 replicates Experiment 1’s finding that people are willing to forgo financial reward to seek information about a delayed outcome when the outcome contingencies are learned via sampling. This preference for information also extends to situations where there is no cost to seeking advance information (equal rewards conditions). Surprisingly, we did not observe increased choice for an option that is optimal (compared with the alternative) when it was informative compared with when it was not.

Regarding people’s outcome estimates (obtained both mid- and post-task), we found no consistent evidence to suggest people were systematically over- or under-estimating the amount of reward (points) they received as a function of the informativeness of available cues. While people showed increased choice preference for the target option when it was informative, evidence that participants overestimated the outcome value of this option was fleeting. For example, while post-task repeated estimates suggested participants significantly overestimated the value of the suboptimal option when it was informative (Fig. 10, target suboptimal), there was no commensurate difference observed in the Block 4 repeated estimates. Moreover, when both options resulted in equal rewards, there was no significant difference in participants’ estimates observed between informative and noninformative conditions.

In summary, the effects present were inconsistent and provide little convincing evidence of a systematic bias towards either over- or under-estimating rewards as a function of informativeness. We revisit this issue in the General Discussion.

Analogous to Experiment 1 the outcome estimates obtained correlated with people’s choice preferences in the expected direction; the more rewarding a participant believed an option to be, as inferred by their outcome estimates, the more likely they were to choose it. This result suggests the outcome estimates obtained in Experiment 2 were, to some extent, tapping participants’ representations of an option’s reward value.

## General discussion

In the past decade a wealth of literature has examined and exhibited peoples’ and animals’ preferences for noninstrumental information about rewarding, aversive, and neutral events (e.g., Bennett et al., 2016; Brydevall et al., 2018; Charpentier et al., 2018; Iigaya et al., 2016, 2020; Kobayashi et al., 2019; Liew et al., 2022, 2023; Mechera-Ostrovsky et al., 2023; Zhu et al., 2017), as well as their willingness to pay for such information (e.g., Bennett et al., 2016; Cabrero et al., 2019; Vasconcelos et al., 2015; Zentall & Stagner, 2011). The goal of the current project was to assess whether people are willing to forgo financial reward to seek information about delayed outcomes when outcome contingencies are learned via sampling. In addition, we examined whether this desire for advance information influences people’s ability to learn the true reward structure of an environment.

In Experiment 1, we found participants’ propensity to choose a suboptimal option increased when that option was information-rich (informing participants of the delayed outcome immediately), as opposed to when it was noninformative. This finding, while unsurprising given people’s willingness to explicitly *pay* for advance information in previous work (e.g., Bennett et al., 2016), shows people also forgo reward in exchange for information when outcomes are learned from experience rather than provided in the instructions.

Participants’ single value estimates of the reward outcomes were similar regardless of an option’s informativeness; while people tended to over- or under-estimate an option’s true value (regressing to the mean of both options), there was no observable difference in estimates between informativeness conditions.

Experiment 2 found that in both the suboptimal and equal-reward conditions participants were significantly more likely to choose the target option when it was informative (Find Out Now) as opposed to when it was noninformative. In the optimal condition, however, participants’ choices were not significantly affected by the presence of advance information.

Participants’ average outcome estimates also correlated with their choice preferences during the choice phase—the more rewarding a participant believed an option to be, relative to the alternative, the more they chose it. We are therefore confident that participants’ outcome estimates were tapping their underlying representations of the reward structure.

Despite our measures seemingly providing an assay of underlying value representation, we found no strong evidence to suggest the presence of advance information influenced people’s ability to learn these values. There were however differences in people’s estimates which, while weak, lessen our confidence in inferring that advance information has *no* effect on people’s ability to learn the reward values they experience. For example, participants’ post-task estimates for the suboptimal option in the ‘target suboptimal’ condition were significantly higher when the option was informative (FON), as opposed to noninformative. Similarly, the estimates for the FON option were numerically higher than the noninformative alternative for all but one estimate (see Fig. 9, target optimal). These differences were in the expected direction if advance information, which is itself valued, inflated people’s beliefs about how objectively rewarding (in terms of points) an option is. Conversely, however, we observed an interaction between informativeness condition and option type in people’s in-task estimates for the optimal condition (see Fig. 9, target optimal). In this instance, people’s estimate of the informative target (FON) was less than its noninformative counterpart. In some cases, these results could be taken as evidence that the presence of information positively biased people’s outcome estimates, in others, the informative option appeared to be undervalued; in most instances, however, the results suggest there is no effect at all.

Returning to Fig. 2 from the Introduction, our results therefore suggest there is no relationship (i.e., a red dotted-line in Fig. 2) between people’s representations of the cue-state and outcome-state. While the cue state is valued when informative, this does not appear to obscure people’s representations of the extrinsically rewarding outcome state.

While the evidence for advance information biasing representations was mixed/weak, the clear preference for noninstrumental information observed in both experiments is commensurate with the predictions of several computational accounts. For example, uncertainty aversion accounts (Bennett et al., 2016) and anticipatory accounts (Iigaya et al., 2016, 2020) both predict our participants’ willingness to forgo reward for information.

In previous work, we have examined how uncertainty aversion (e.g., Bennett et al., 2016) and anticipation-based (e.g., Iigaya et al., 2016) models compare in capturing data from a secrets task in which outcomes and probabilities are described (see Liew et al., 2022). Here, however, we refrain from fitting these models because they are not explicitly concerned with the representations of the outcome state, thus making them less applicable for shedding light on our current focus. We provide a more detailed explanation of the models and the limits of their usefulness for the current data in the Supplementary Materials.

The current work also provides evidence that the rewards people receive on a trial influence their subsequent choices (see the win-stay, lose-shift model analysis), as observed in similar bandit tasks (e.g., Worthy et al., 2013). People’s win-stay, lose-shift behaviours did not differ however between information conditions; this suggests that while people showed increased preferences for informative options, their sensitivity to the previously experienced outcome was unaltered by the presence of noninstrumental information.

## Limitations and future directions

Although the cues we used in our Informative conditions were informative in the sense that they signalled either ‘wins’ or ‘no-wins’ (i.e., smiley and sad faces after choosing Find Out Now), it is worth noting these cues did not entirely resolve uncertainty about reward outcomes. When choosing Find Out Now, a no-win cue signalled zero points with 100% certainty; however, if participants received a cue signalling a win, there still remained uncertainty as to how many points would be won (as points were uniformly distributed within a 20-point range). These sampling distributions were used so the average reward values associated with both options were not obvious to participants, but this may have unintentionally reduced the perceived value of the Find Out Now option as it did not resolve all uncertainty.

Relatedly, although cartoon faces have been used as cues in past work to signal delayed outcomes (e.g., Liew et al., 2022) they may hold intrinsic social value which evoked additional affective responses. We chose these cues so that participants learned which cues signalled reward (as opposed to no-reward) relatively quickly, but this may have had unintended effects on participants’ choices and outcome learning. It is however worth noting that previous work using faces as cues (e.g., Liew et al., 2022, 2023) has replicated the effects of temporal delay and reward probability on people’s choice preferences observed in tasks which used neutral cues (Iigaya et al., 2016, 2020).

A further consideration was the high variance in responses across our types of memory estimate probes. Many participants gave odd responses (e.g., outcome values they never saw) as well as responses they were explicitly asked not to give (e.g., zero points when asked to estimate the outcome of a win trial). Some of this variance may be due to participants misunderstanding the instructions (such as believing we were asking for estimates of total points earned), but it is difficult to demarcate participants who have misunderstood instructions from those who have unusual beliefs about how rewarding the options were. In our analysis we opted for an exclusion criterion that screened out extreme estimates (e.g., those above 1,000) but this likely came at the price of including some participants who did not fully understand the instructions and gave responses that did not align with the representations we were aiming to probe. We present additional analyses in the Supplementary Materials which explore the impact of different exclusion criteria.

Future research investigating the role of advance information on people’s representations of reward should consider how to better probe such representations. One possible method is to incentivize participants’ estimates such that they earn more money the closer their estimate is to the true value. Another possible approach is to explicitly tell participants they will be required to answer questions about reward values during the instruction phase. We did not opt for such a strategy here as we did not want to bias choice—as making participants pay closer attention to the point values received may affect their choices—but such a method may lead to more accurate responding.

## Conclusion

The results of these two experiments further support the idea humans intrinsically value information, even when the information available has no obvious instrumental use. Here, we find that people are willing to forgo financial reward to obtain advance information about delayed outcomes in a setting where contingencies, and therefore costs, are learned from experience rather than described to participants. Overall, our findings did not suggest that an inaccurate representation of learned reward structure drives a preference for noninstrumental, advance information, although further research is warranted in order to enable stronger conclusions to be drawn.

### Supplementary Information

Below is the link to the electronic supplementary material.

Supplementary file1 (DOCX 1676 KB)

## Footnotes

1 Sample sizes for all three experiments never exceeded more than 17 subjects per between-subjects cell. Furthermore, in Molet et al. (2012) and McDevitt et al. (2019) the ‘informative’ nature of the suboptimal option (which resulted in reward, 10 points, 20% of the time, yet signalled the delayed outcome) did not resolve uncertainty more than the optimal option (which resulted in reward, 3 points, 100% of the time, yet was ‘unsignalled’). If it is resolving uncertainty (or anticipating delayed rewards) which drives noninstrumental information seeking, it is unsurprising no preference was observed in such tasks as the outcome is not uncertain for either option.

2 Using Fig. 1 as an example, IPEs would occur when the reward-predictive cues are displayed (cue state) and RPEs occur when the actual reward is delivered (outcome state).

3 mTurk has come under scrutiny (Webb & Tangney, 2022) for having a large number of ‘bots’ as well as participants who are completing multiple HITs (i.e., online tasks) simultaneously. While our experiments may not have been affected by this issue—and we imposed an exclusion criterion based on a multi-item attention check questionnaire—we err on the side of caution in terms of drawing strong conclusions given this possibility.

4 We report the results of an analysis where we rerandomize the allocation of target and non-target to either square or triangle for the noninformative, equal rewards condition in the Supplementary Materials. The main effect of informativeness is robust against this rerandomization.
