# Supplementary material for: The effect of noninstrumental information on reward learning
Source: Mem Cognit. 2024 Feb 23;52(5):1210–27. doi: 10.3758/s13421-024-01537-4 (PMC11315740; doi:10.3758/s13421-024-01537-4)
Supplement: Supplementary file 1 — Supplementary file1 (DOCX 1676 KB) [file 13421_2024_1537_MOESM1_ESM.docx]

**Supplementary Materials**

This document contains the appendices for Embrey, Li, Liew, & Newell “The Effect of Non-Instrumental Information on Reward Learning”. It contains the following five sections:

1. Attention check quiz details
2. Experiment 1: Effect of participant removal
3. Experiment 2: Effect of participant removal
4. Single-value estimates in Experiment 2
5. Re-randomising non-informative, equal rewards target and non-target
6. Relationships between estimates and choice
7. Computational models

**Section 1 - Attention check quiz details**

Both experiments had a three-item quiz following the instructions, and before the main task started, to ensure participants had paid attention.

Question 1 was “How long is the delay?” with four possible answers (10 seconds, 20 seconds, 30 seconds, 40 seconds). Question 2 was a true or false question: “There is a 50% chance of winning points and a 50% chance of 0 points on each trial". Question 3 was another true or false question: “Choosing 'Keep It Secret' will always lead to a 'Confused Face' cue".

Participants had to get all three questions right to continue. If they got one or more questions wrong, the task sent the participant back to the beginning of the instructions to re-read them, following which the participant had to re-complete the attention check.

**Section 2 – Effect of Participant Removal: Experiment 1**

***Post-Test Single Value Estimates***

In the main manuscript we report the results of an analysis where we removed participants who gave improbable estimates greater than 1,000, as well as any estimates of value 0 (which they were explicitly asked not to give). Recall that we ran a two-way mixed ANOVA on participant estimates, with option type (optimal vs. sub-optimal) as within-subjects factor and informativeness condition (informative vs. non-informative) as between-subjects factor. In the following we report this same analysis in two scenarios with different exclusion criteria. In Scenario 1, no exclusion criteria are applied. In Scenario 2, we impose a stricter exclusion criterion.

**Scenario 1: No Exclusion Criteria.** Consider the first scenario where no participants are removed (i.e., *N* = 99). Participant estimates with no exclusion criteria are illustrated in Figure S1.

**Figure S2a**

*Participants’ post-test estimates of mean observed outcome values of “win trials” in Experiment 1, given no exclusion criteria were applied.*

**
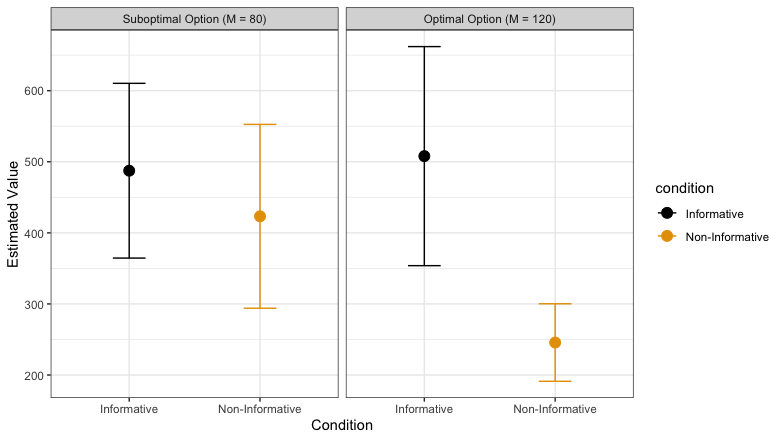
**

KIS

FON

*Note*. Labels FON and KIS denote the Find Out Now and Keep It Secret Options for informative conditions. Error bars denote ±1 standard error of the mean.

With no exclusion criteria, we added an extremely small constant to estimates (1.0x10^18^) prior to log-transforming them as we had done in the main manuscript, as some estimates had a value of 0 and would thus lead to -Inf when log-transformed. Here, we did not find a significant difference in post-test estimates between the informative vs. non-informative conditions, *F*(1, 97) = 0.978, *p*  = .325, η^2^ = .005, nor a main effect of option type, *F*(1, 97) = 1.770, *p* = .186, η^2^ = .009. There was no significant interaction effect between informativeness condition and option type, *F*(1, 97) = 2.090, *p* = .152, η^2^ = .010.

Here, for the suboptimal option, *M* = 487.43 for informative condition vs. *M* = 423.27 for non-informative condition; and for the optimal option, *M* = 507.94 for informative condition vs. *M* = 245.71 for non-informative condition. Note the extremely high mean estimates in the above due to participants giving estimates in the 1000s; this is presumably due to participants misunderstanding the instruction and giving estimates of the *total* points they would win over the whole task, rather than on a single trial.

**Scenario 2: Strict Exclusion Criteria.** We also conducted an analysis with an especially strict exclusion criterion. Here, we removed participants who gave responses they did not observe in the task (i.e., values less and 70 or greater than 130 points) in addition to removing participants who gave 0 as an estimate. These criteria resulted in the removal of 56 participants, and thus a remaining sample of 43 (*n =* 21 informative condition; *n* = 27 non-informative condition). Participant estimates with these strict exclusion criteria are illustrated in Figure S2.

**Figure S2b**

*Participants’ post-test estimates of mean observed outcome values of “win trials” in Experiment 1, given strict exclusion criteria were applied.*


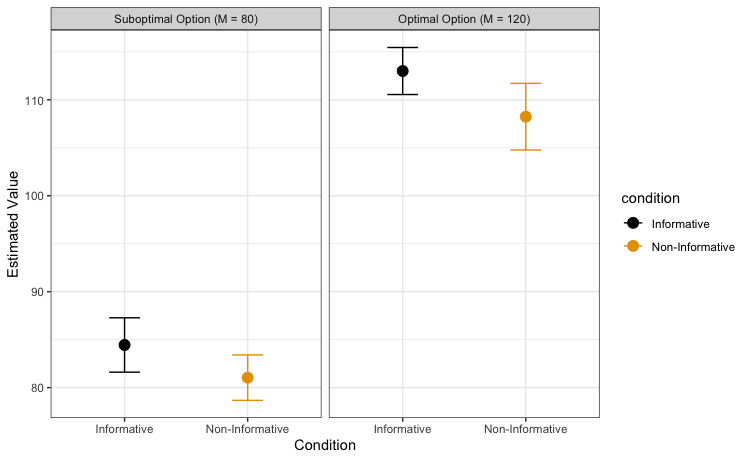


KIS

FON

*Note*. Labels FON and KIS denote the Find Out Now and Keep It Secret Options for informative conditions. Error bars denote ±1 standard error of the mean.

Again, estimates were log-transformed prior to analysis. With these strict exclusion criteria, we did not find a significant difference in post-test estimates between the informative vs. non-informative conditions, *F*(1, 41) = 2.111, *p*  = .154, η^2^ = .028. However, we found a main effect of option type, *F*(1, 41) = 102.35, *p* < .001, η^2^ = .523. There was no significant interaction effect between informativeness condition and option type, *F*(1, 41) = 0.046, *p* = .830, η^2^ = .0005. Hence, the same effects were found as with the exclusion criteria used in the manuscript.

Here, for estimates of the suboptimal option, *M* = 84.52 for informative condition vs. *M* = 81.70 for non-informative condition; and for the optimal option, *M* = 113.31 for informative condition vs. *M* = 107.66 for non-informative condition.

**Section 3 – Effect of Participant Removal: Experiment 2**

In the main manuscript we report the results of people’s value estimates where we removed participants reporting improbable values greater than 1000, as well as trials where people reported 0. Below we report the same analyses for two different exclusion criteria. One where no datapoints were removed, and another where a strict exclusion criteria was used. We report the results for both types of estimates reported in the main manuscript (i.e., in-task and post task estimates).

***In-Task (Block 4) Estimates***

**Scenario 1: No Exclusion Criteria.** Firstly, we report the analyses where no participants were removed. Participant estimates with no exclusion criteria are illustrated in Figure S3. The following analyses are all linear mixed models with option type (target vs. non-target), informativeness condition, and their interaction term as fixed effects, and with random intercepts for subjects.

**Figure S3a**

*Participants’ in-task estimates of outcome values for “win trials” during Block 4 in Experiment 2, with no exclusion criteria.*


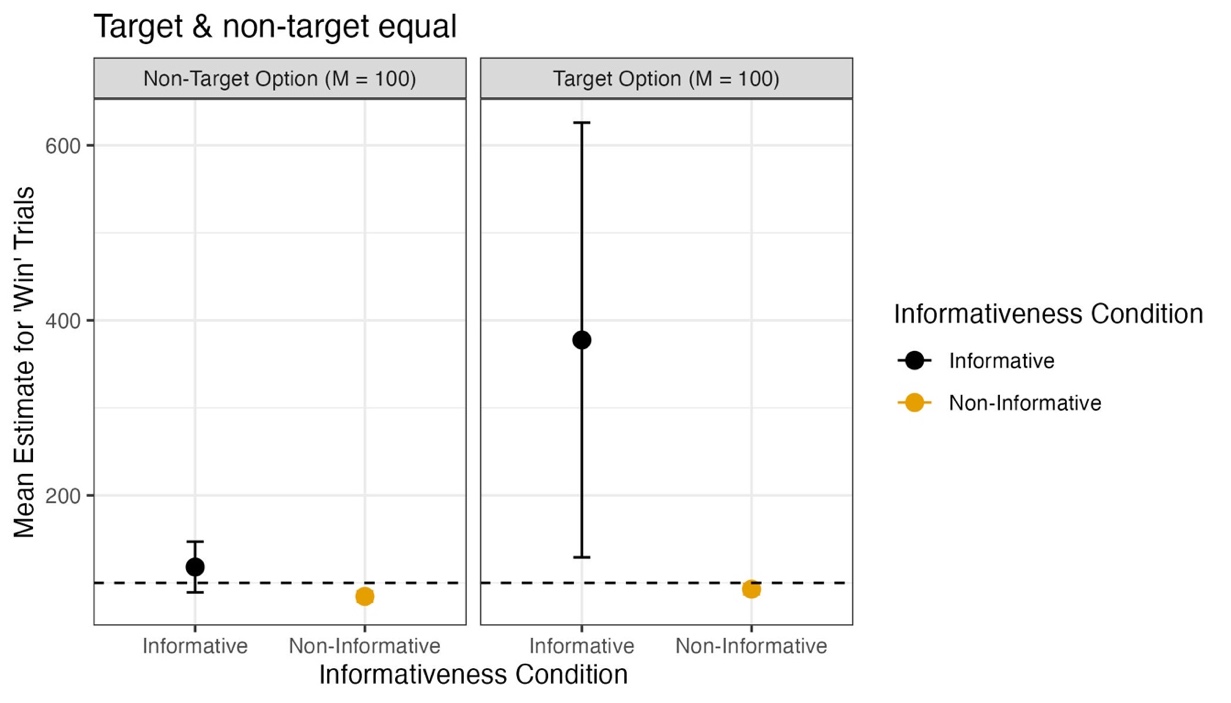


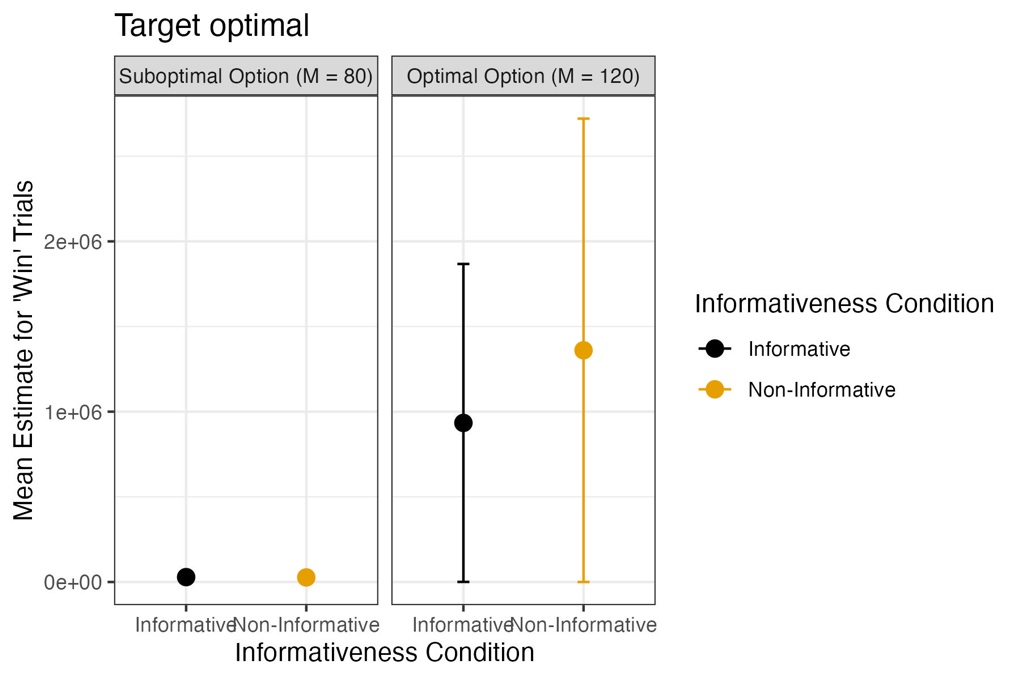

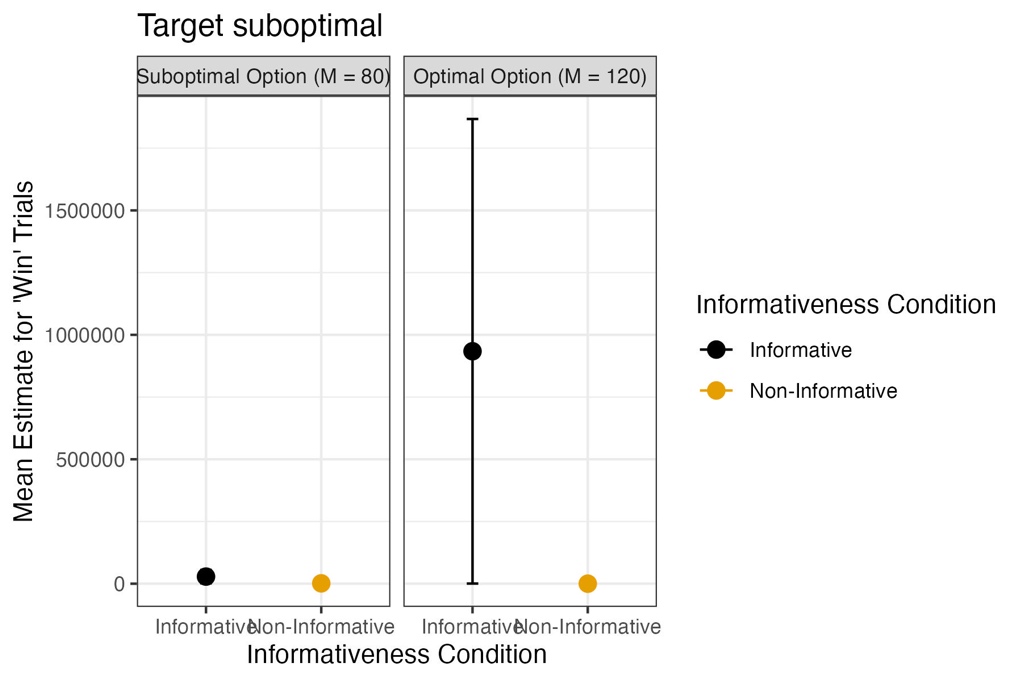


FON

KIS

KIS

FON

*Note.* Top panels depict mean estimates from block 4 from the *target and non-target equal* optimality condition; bottom panels depict those from the *target optimal* and *target suboptimal* conditions. In the bottom panel, data from the control group (*target and non-target unequal, noninformative* condition) is shown twice; first as *target optimal, non-informative*, and second as *target suboptimal, non-informative.* Find Out Now (FON) and Keep It Secret options for the informative condition are labelled as such. Error bars denote ±1 standard error of the mean. Dashed lines denote true observed mean value, but omitted for bottom two panels due to scale of y-axis.

When the target and non-target options were equal in average reward, we observed no effect of the informativeness condition (*p =* .856), option type (*p* = .150), nor the interaction between the two (*p* = .337).

In conditions where the target was optimal, we observed no significant effect of informativeness condition (*p* = .999), option type (*p* = .435), nor their interaction (*p* = .793).

In conditions where the target was sub-optimal, we similarly observed no significant effect of informativeness condition (*p* = .158), option type (*p* = .174), nor their interaction (*p* = .334).

**Scenario 2: Strict Exclusion Criteria.** Next, we conducted the same analyses where we enforced a strict exclusion criterion. First, we removed participants who gave a response they could not have observed (this differs by condition) (*n=* 192)– instead of only single responses >= 1000. Second, we removed participants who gave ‘0’ responses which they were explicitly asked not to give (*n* = 116) – instead of removing only *instances* of these responses. Together, 192 out of 253 participants were removed from analysis, resulting in a sample size of 61. Participant estimates following these strict exclusion criteria are illustrated in Figure S4.

**Figure S3b**

*Participants’ in-task estimates of outcome values for “win trials” during Block 4 in Experiment 2, with strict exclusion criteria.*


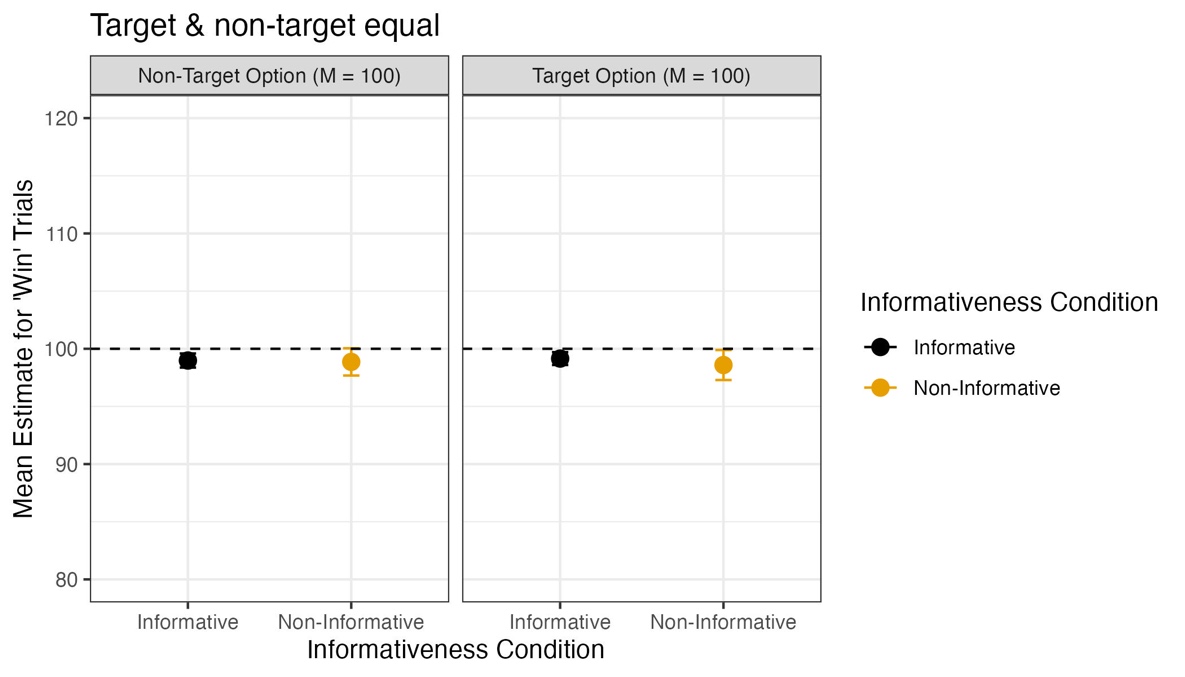


FON

KIS


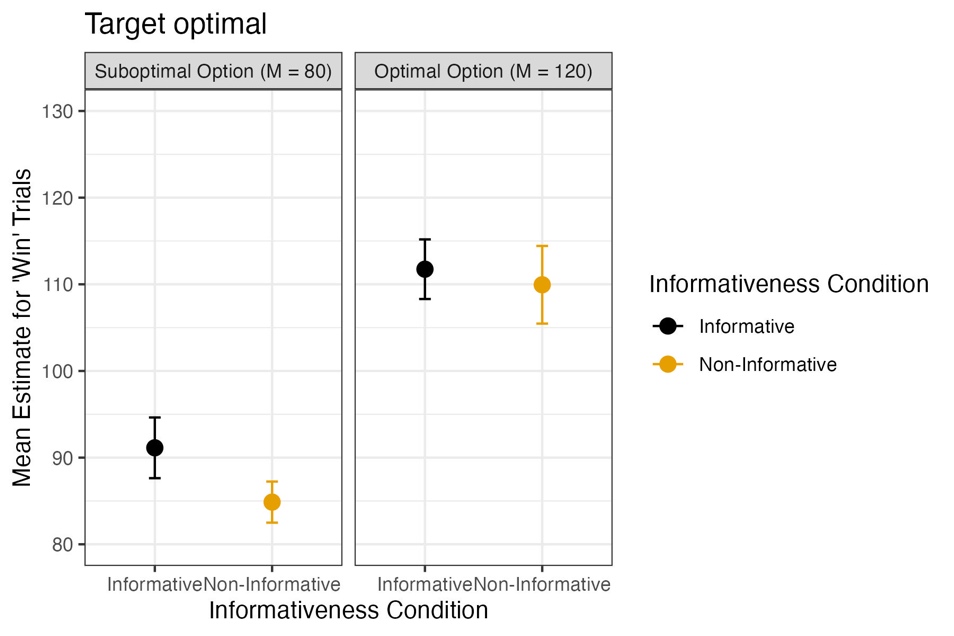

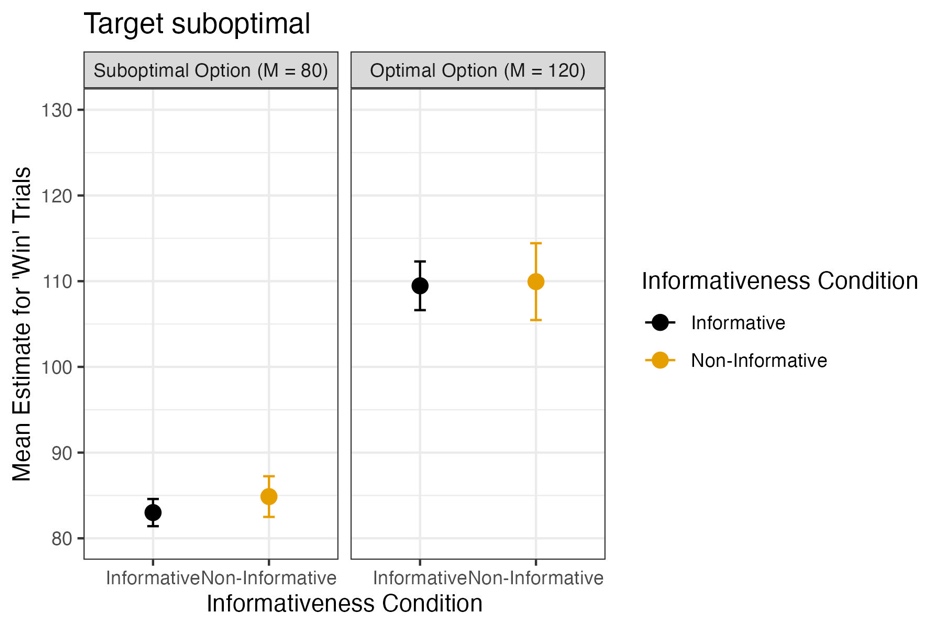


KIS

FON

FON

KIS

*Note.* Top panels depict mean estimates from block 4 from the *target and non-target equal* optimality condition; bottom panels depict those from the *target optimal* and *target suboptimal* conditions. In the bottom panel, data from the control group (*target and non-target unequal, noninformative* condition) is shown twice; first as *target optimal, non-informative*, and second as *target suboptimal, non-informative.* Find Out Now (FON) and Keep It Secret options for the informative condition are labelled as such. Error bars denote ±1 standard error of the mean. Dashed lines denote true observed mean value.

When the target and non-target options were equal in average reward, we observed no effect of the informativeness condition (*p =* .894), option type (*p* = .526), nor the interaction between the two (*p* = .459).

In conditions where the target was optimal, we observed no significant effect of informativeness condition (*p* = .105). However, there was a significant effect of option type (*β* = 25.08, *t*(696) = 17.176, *p* < .001), and a significant interaction between informativeness condition and option type (*β* = -4.47, *t*(696) = -2.338, *p* = .020).

In conditions where the target was sub-optimal, we observed no significant effect of informativeness condition (*p* = .851), nor of the interaction between informativeness condition and option type (*p* = .464). There was a significant effect of option type (*β* = -25.08, *t*(638) = -18.025, *p* < .001).

***Post-Test Repeated Value Estimates***

**Scenario 1: No Exclusion Criteria.** First, we report the analyses where no participants were removed. Post-test repeated value estimates following no exclusion criteria are illustrated in Figure S5.

**Figure S3c**

*Participants’ mean repeated estimates of the mean observed outcome values across both “win trials” and “no-win trials” in Experiment 2, with no participants excluded.*


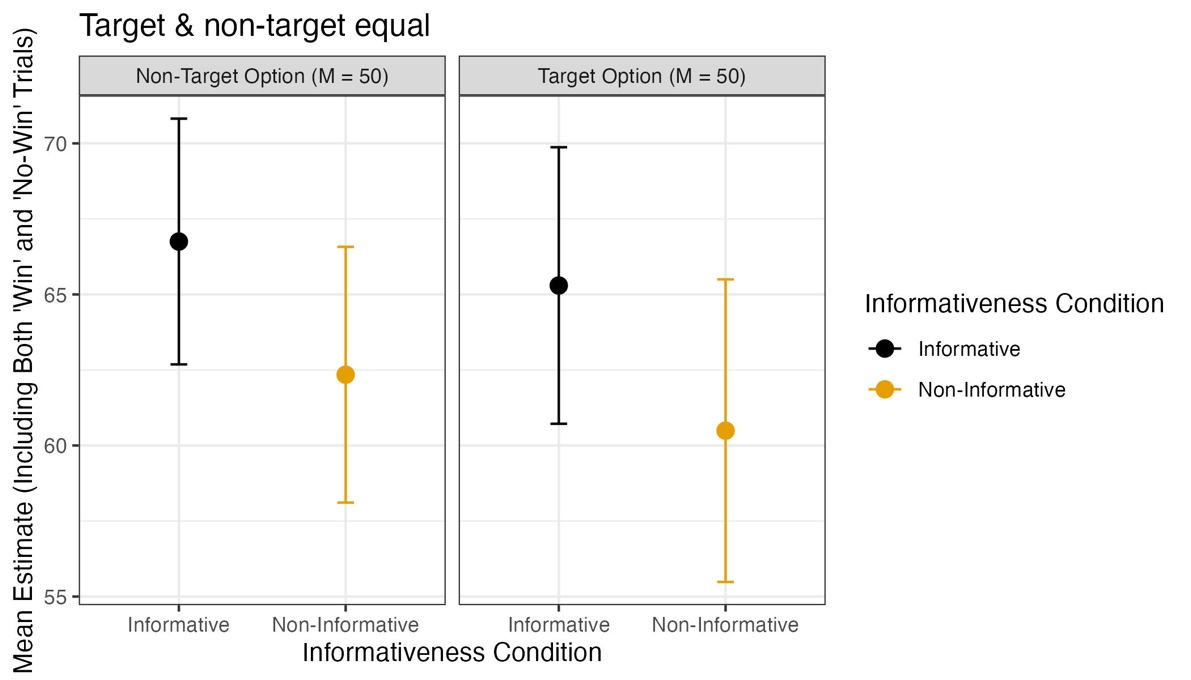


FON

KIS

**
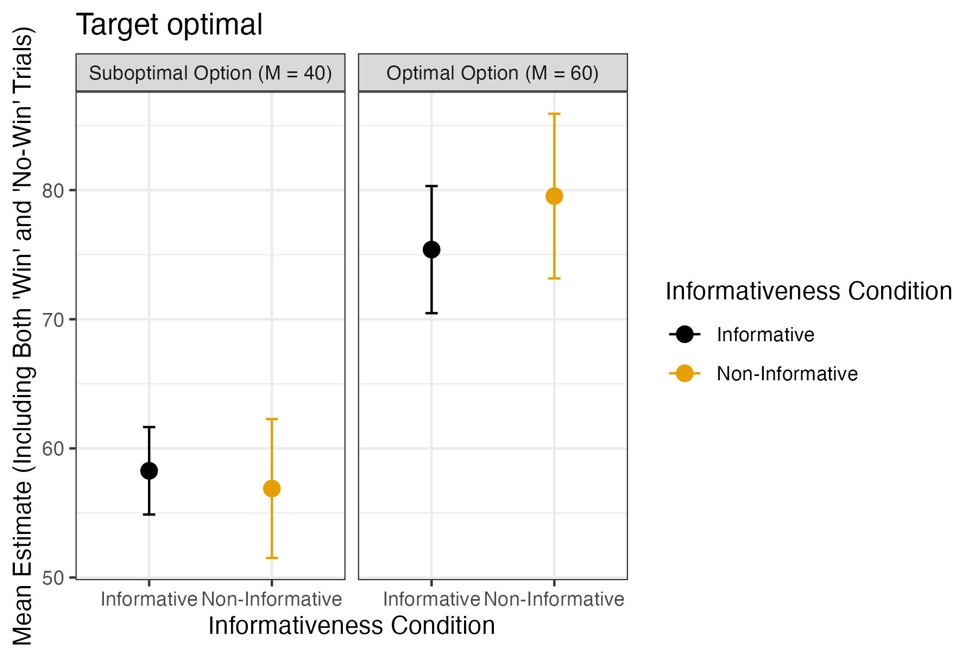

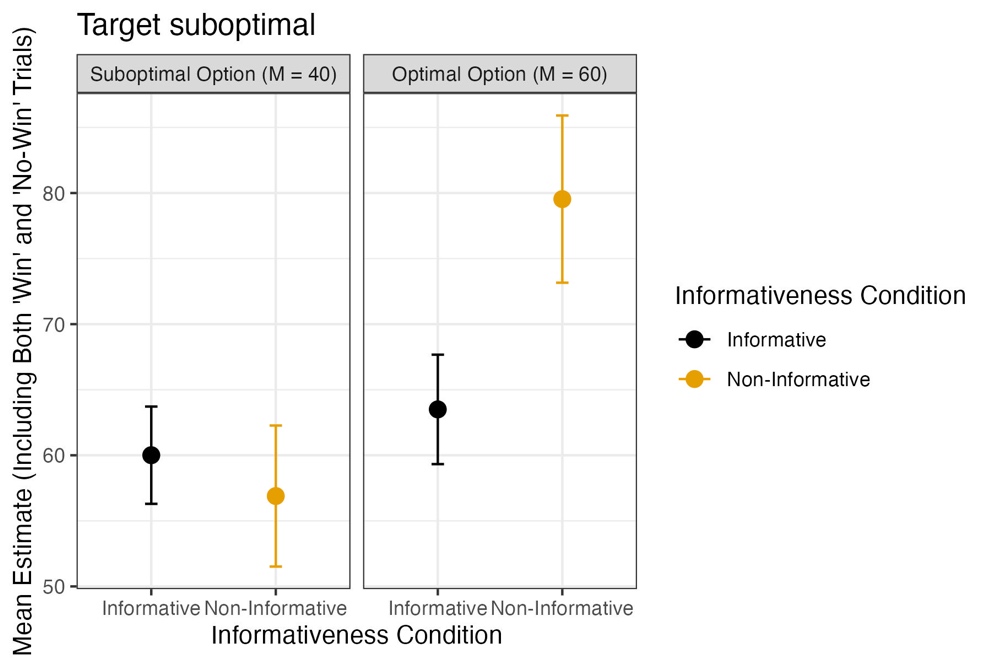
**

KIS

FON

FON

KIS

*Note.* Participants means are obtained from averaging across their 10 repeated estimates provided for each of the two choice options. In the bottom two panels, data from the control group (*target and non-target unequal, noninformative* condition) is shown twice; first as *target optimal, non-informative*, and second as *target suboptimal, non-informative.* Find Out Now (FON) and Keep It Secret options for the informative condition are labelled as such. Error bars denote ±1 standard error of the mean.

When the target and non-target options were equal in average reward, we observed no effect of the informativeness condition (*p =* .462), option type (*p* = .702), nor the interaction between the two (*p* = .940).

In conditions where the target was optimal, we observed no significant effect of informativeness condition (*p* = .953) nor of the interaction between informativeness condition and option type (*p* = .481). However, there was a significant effect of option type (*β* = 17.128, *t*(1889.88) = 4.593, *p* < .001).

In conditions where the target was sub-optimal, we observed a significant effect of informativeness condition (*β* = 16.036, *t*(145.879) = 2.352, *p* = .020), such that estimates were higher in the non-informative condition compared to the informative condition. There was also a significant effect of the interaction between informativeness condition and option type (*β* = -17.42, *t*(1895.329) = -3.04, *p* = .002). There was no significant effect of option type (*p* = .387).

**Scenario 2: Strict Exclusion Criteria.** Next, we conducted the same analyses where we enforced a strict exclusion criterion. First, we removed participants who gave a response they could not have observed (this differs by condition) (n = 126)– instead of only estimates of >= 1000. Second, identical to what was report in the manuscript, we excluded participants who did not report both 0s *and* non-0 estimates for either of the two observed choice options as they were instructed to do (n = 91). These criteria resulted in the exclusion of 163 participants, and a remaining sample of 90 participants. Post-test repeated estimates following these strict exclusion criteria are illustrated in Figure S6.

**Figure S3d**

*Participants’ mean repeated estimates of the mean observed outcome values across both “win trials” and “no-win trials” in Experiment 2, with strict exclusion criteria.*


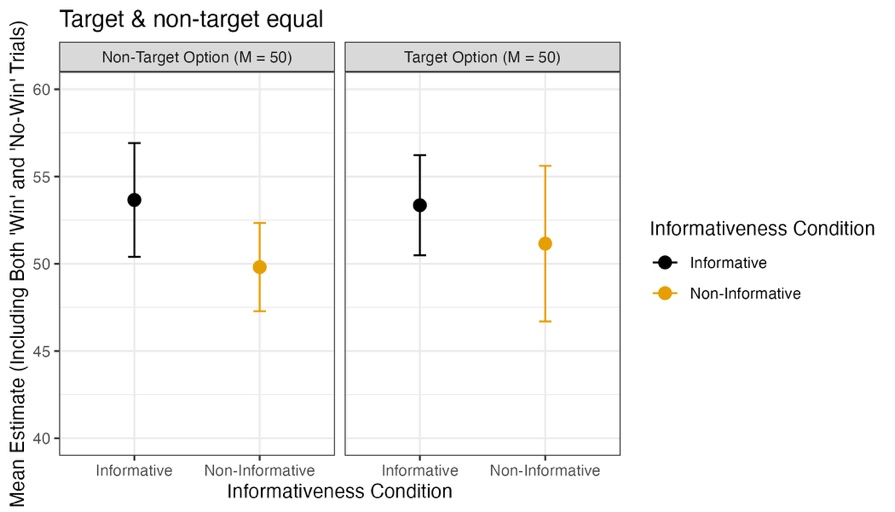


FON

KIS

KIS


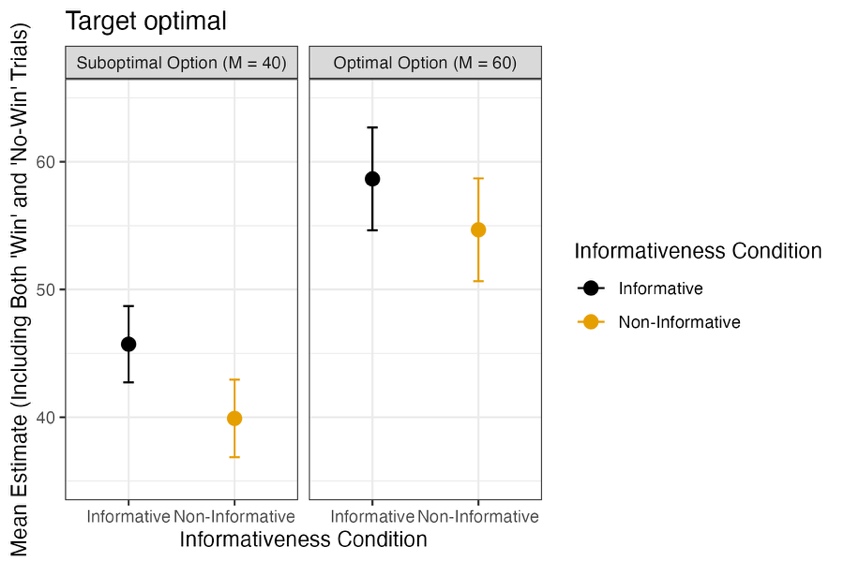

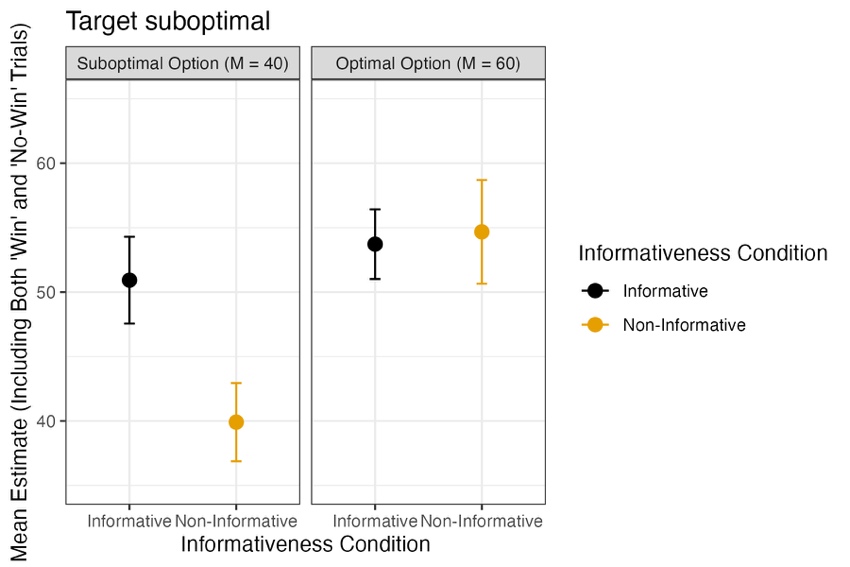


FON

FON

KIS

*Note.* Participants means are obtained from averaging across their 10 repeated estimates provided for each of the two choice options. In the bottom two panels, data from the control group (*target and non-target unequal, noninformative* condition) is shown twice; first as *target optimal, non-informative*, and second as *target suboptimal, non-informative.* Find Out Now (FON) and Keep It Secret options for the informative condition are labelled as such. Error bars denote ±1 standard error of the mean.

When the target and non-target options were equal in average reward, we observed no effect of the informativeness condition (*p =* .359), option type (*p* = .718), nor the interaction between the two (*p* = .897).

In conditions where the target was optimal, we observed no significant effect of informativeness condition (*p* = .561) nor of the interaction between informativeness condition and option type (*p* = .748). However, there was a significant effect of option type (*β* = 12.118, *t*(700) = 2.198, *p* = .028).

In conditions where the target was sub-optimal, we observed no significant effect of informativeness condition (*p* = .614), option type (*p* = .066), nor the interaction term (*p* = .483).

**Section 4 – Post-Test Single Value Estimates, Experiment 2**

In this section we report the results and analysis for single value estimates obtained in Experiment 2. These single value estimates were obtained in the same manner as Experiment 1. We report them here as opposed to the main text as we believe the added in- and post-task repeated estimates provided a better probe of people’s mental representations of the outcomes.

To exclude improbable final estimates, point estimates of 0 or more than 1000 were excluded from visualisation and analyses. This led to the exclusion of 8.50% of the total data. Estimates from participants following exclusion are shown in Figure S7.

**Figure S4a**

*Participants’ post-test estimates of mean observed outcome values of “win trials” in Experiment 2.*


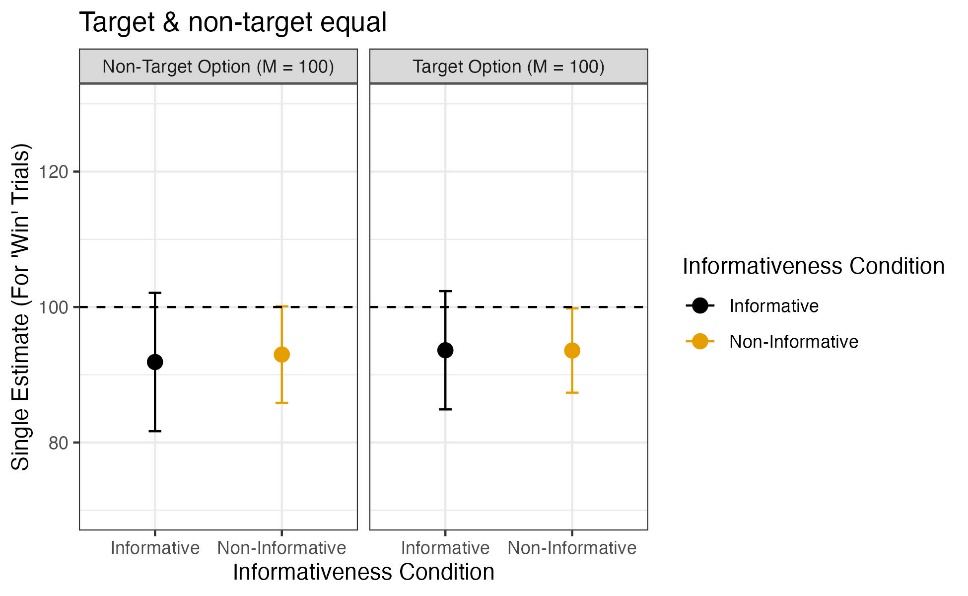

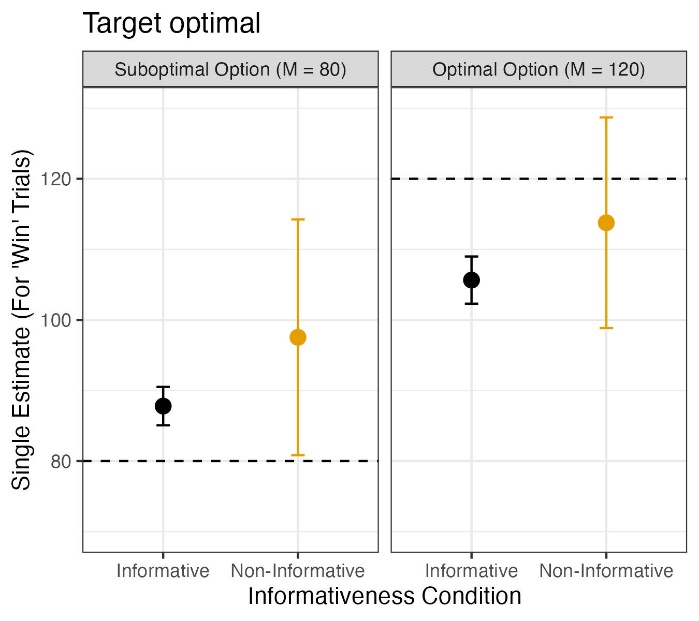

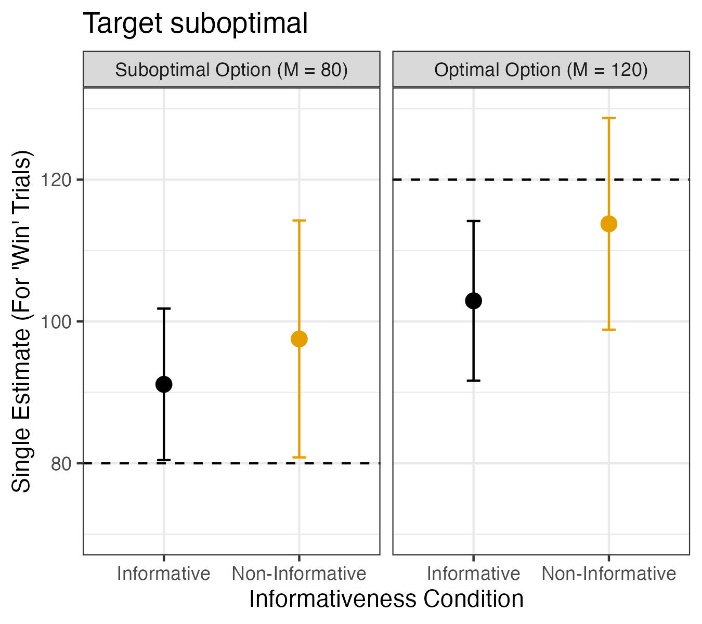


KIS

KIS

KIS

FON

FON

FON

*Note.* In the bottom two panels, data from the control group (*target and non-target unequal, noninformative* condition) is shown twice; first as *target optimal, non-informative*, and second as *target suboptimal, non-informative.* Error bars denote ±1 standard error from the mean. Dashed lines denote true observed mean value.

We first analysed participants’ final estimates from the *target and non-target equal* optimality condition. A linear mixed-effects model was fitted with each participant estimate as the dependent variable, informativeness condition, option type, and their interaction term as predictors, and subject as random variable. None of the predictors were found to be significant (*informativeness condition*: *p* = .379; *option type*: *p* = .739; *interaction* *term*: *p* = .816).

When the target option was associated with optimal outcomes compared to the non-target option, *t-*tests revealed that neither estimates for the suboptimal nor optimal option differed significantly between informativeness conditions (for the suboptimal option: *p* = .504, *M* = 80.15 for informative vs. *M* = 91.17 for non-informative; for the optimal option: *p* = .430, *M* = 96.98 for informative vs. *M* = 109.02 for non-informative).

When the target option was associated with suboptimal outcomes compared to the non-target option, *t-*tests revealed that neither estimates for the suboptimal nor optimal option differed significantly between informativeness conditions (for the suboptimal option: *p* = .841, *M* = 87.33 for informative vs. *M* = 91.17 for non-informative; for the optimal option: *p* = .655, *M* = 100.96 for informative vs. *M* = 109.02 for non-informative).

**Section 5 – Relationship between estimates and choice**

Below are the figures for the relationship between people’s estimates and their choices in both Experiments 1 and 2.

**Experiment 1**

Below is the figure plotting the relationship between people’s single value estimates obtained after the sampling phase and their choices in the task. The data analysed (in the main manuscript) and presented below is that of the participants who met the inclusion criteria (i.e., estimates < 1000 and greater than 0). In addition, we have removed three (3) outlier participants from the below plot as they gave estimates greater than ±200. Simply, the figure is uninformative when these points are included as the majority of data is clumped in the centre as a result of the x-axis’ expansion.

We also note that while the analysis in the main manuscript was averaged across conditions, we present the data per group to highlight the consistency of the trend irrespective of condition.

**Figure S5a**

*Relationship between people’s single value estimates and their choices for Experiment 1*


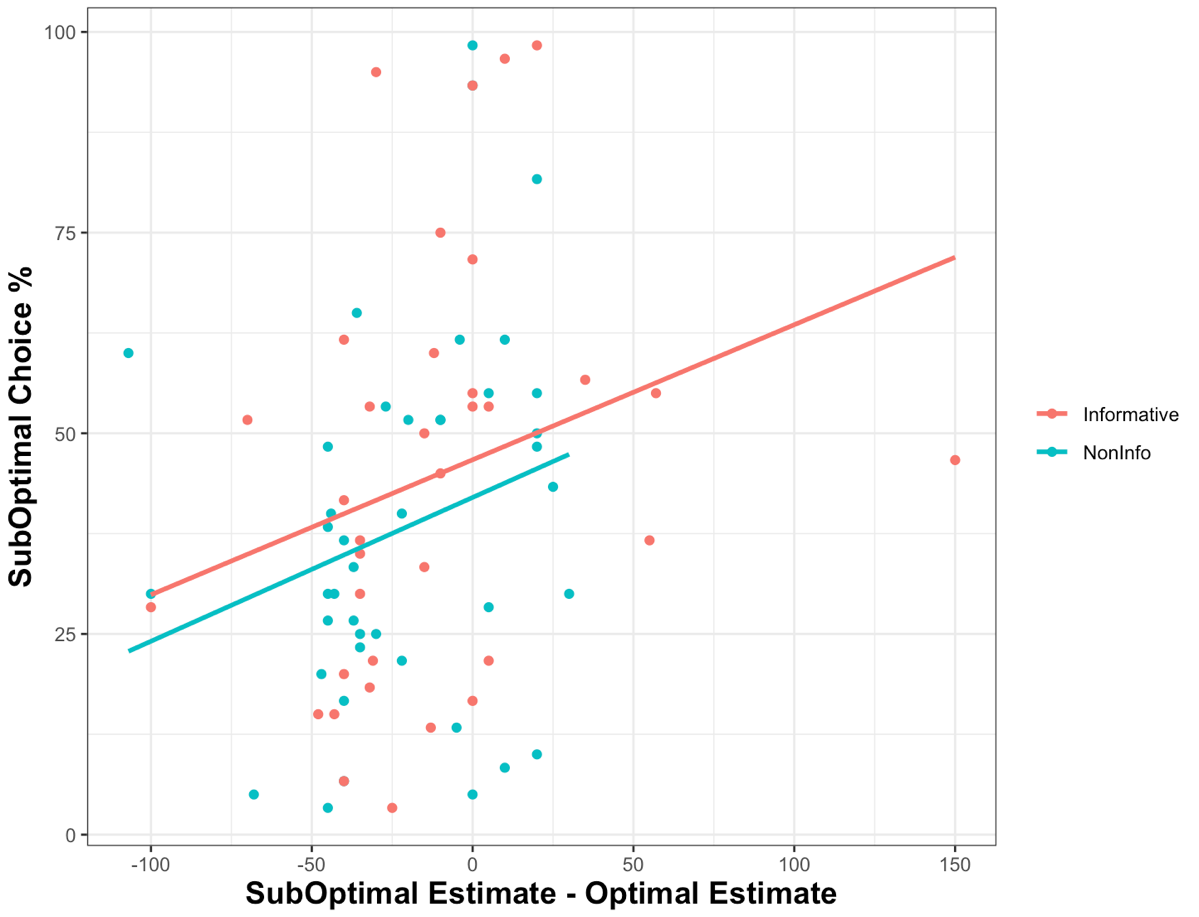


Note. For aesthetics’ sake, the data presented above is sans datapoints where the difference in people’s estimates is ±200.

**Experiment 2**

***Block 4 Estimates***

Below is the figure depicting the relationship between people’s choice preferences in the task and their average estimates made in block 4. The data plotted below is that used for the main block 4 analysis. That is, estimates greater or equal to 1000 were removed and all 0-point estimates were also removed. In total, 18.10% of the data were excluded.

The linear regression model run in the main manuscript was averaged across conditions. We however plot the data differentiated by condition below.

**Figure S5b**

*Relationship between peoples’ estimates in block 4 and their choice preferences in the task.*

*
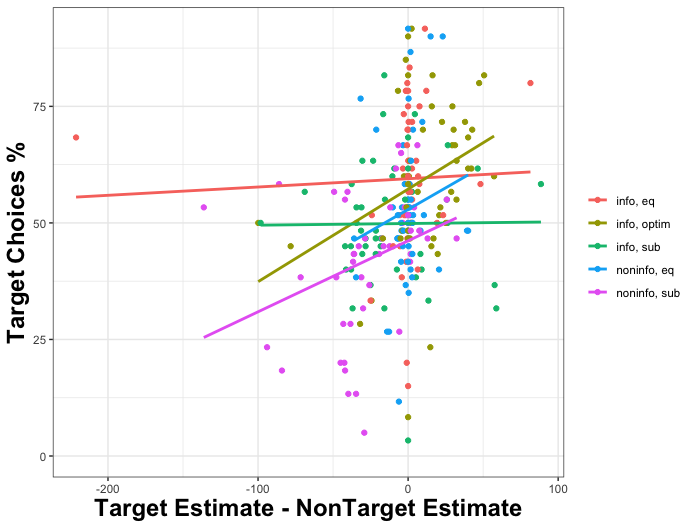
*

***Post Task Estimates***

Below is the figure depicting the relationship between people’s repeated estimates made post task and their choices in the sampling phase. The participants included in the analysis were the same as those included in the analysis of repeated post-task estimates in the main manuscript. Estimates greater or equal to 1000 were removed, as well as participants who did not make both zero and non-zero estimates. This led to a remaining sample size of 164 in the analysis.

While the linear regression run in the main analysis was run across conditions, we report the data below differentiated by condition to show the relationship is generally consistent across conditions.

**Figure S5c**

*The relationship between participant’s post-task repeated estimates and their choices during the task*


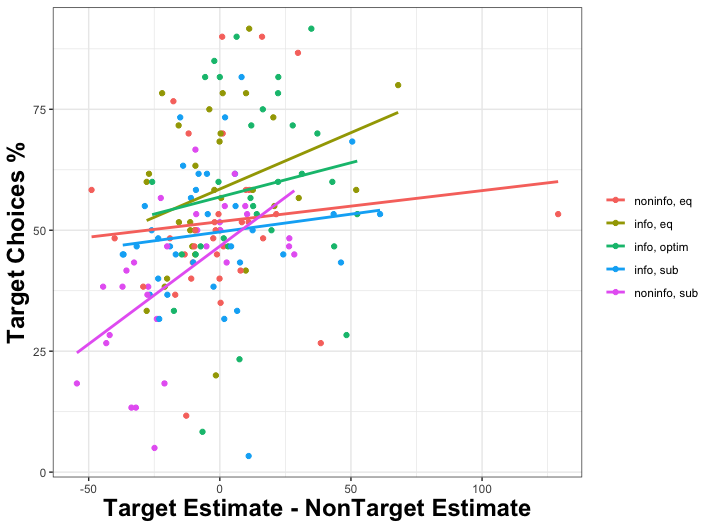


**Section 6 – Re-randomising target and non-target for non-informative, equal rewards**

As noted in the main manuscript, the allocation of ‘target’ and ‘non-target’ to either the square or triangle in the equal rewards, non-informative condition was arbitrary since both options, on average, result in ~50 points.

To check the robustness of our results we re-randomised the allocation of target and non-target and conducted the same analyses as reported in the main manuscript. The effect of informativeness was qualitatively the same as the original randomisation, but the effect of bandit trial was no longer significant. We summarise the relevant stats for this re-randomisation below.

The informativeness of the cues (OR = exp($\beta)$ = 1.46, 95% CI: [1.11, 1.93], *z* = 2.67, *p*  = .007) was a significant predictor of choice with those in the Informative condition choosing the target more than those in the Non-Informative condition. Trial number was no longer significant predictor of choice with choices for the optimal option increasing across trials (*p* = .097). A more complex model incorporating an interaction term between condition and trial was not significantly different to the simpler model (χ² (1) = 3.435, *p* =.064).

**Section 7 – Computational Models**

There are several computational models which could be used directly (or slightly adapted) to fit the data from the Experiments reported in the main manuscript. Broadly, these models fall into two categories: those which posit uncertainty-aversion to drive information seeking (e.g., Bennett et al., 2016) and those which argue anticipatory savouring of future rewards drives information seeking (e.g., Iigaya et al., 2016). As stated in the main manuscript, however, these models do not specify any direct relationship between people’s representations of the cue state and the outcome state. Simply, the participants’ encoding of the objective reward received (i.e., points in our Experiments) is determined by the value of the reward alone or the discounted value of that reward (due to it being delayed), depending on the model. While not exhaustive, we briefly detail two models to bolster this point: the Uncertainty Penalty Model (Bennett et al., 2016) and the Reward Prediction Error with Anticipation model (Iigaya et al., 2016).

***Uncertainty Penalty Model***

The crux of the Uncertainty Penalty model (Bennett et al., 2016) is concerned with how agents determine the subjective value of a given state in the task. The states in our task were the choice state, cue state, and outcome state. As state transitions are beyond the individuals control once a choice (FON or KIS; square or triangle) has been made, the primary values of interest are those ascribed to the options themselves: FON and KIS, or, square and triangle. which is given by

$$\begin{aligned} V\left( s \right)= \sum_{s^{'}} P\left( s^{'} | s \right)\left( R_{\dagger}\left( s, s^{'} \right)+V\left( s^{'} \right)e^{-kU\left( s^{'} \right)} \right)\#\left( 1 \right) \end{aligned}$$

where $P\left( s^{'} | s \right)$ is the probability the next state will be $s^{'}$, given the current state, $s$. For example, if the current state was the FON option, $P\left( s^{'} | s \right)$ denotes the probability the next state will be a smiley face/sad face. $R_{\dagger}\left( s, s^{'} \right)$refers to the objective reward received in the next state. This term is equivalent to zero unless the subsequent state is the outcome state where points are received. The remainder of the equation is concerned with the value of the next state $V\left( s^{'} \right)$, the amount of uncertainty associated with the next state $U\left( s^{'} \right)$, and the free parameter, *k*, which scales an individual’s aversion to uncertainty. Uncertainty in any given state is defined by

$$\begin{aligned} U\left( s \right)=-P\left( s_{w} | s \right){log}_{2}P\left( s_{w} | s \right)-P\left( s_{l} | s \right){log}_{2}\left( s_{l} | s \right)\#\left( 2 \right) \end{aligned}$$

Where $P\left( s_{w} \right| s)$ and $P\left( s_{l} \right| s)$ refer to the probability of either winning points or losing points. In our experiments the probability of a win or loss was always 0.5^[[1]](#footnote-1)^.

Importantly, this model does not specify any mechanism for how the reward at the outcome state, $R_{\dagger}\left( s, s^{'} \right)$, is encoded or represented.

***Reward Prediction Error with Anticipation***

This model, henceforth RPE-A, was developed by Iigaya et al., (2016) and posits that information seeking is driven by an agent’s positive anticipation of future rewards (i.e., savouring). The computational model involves many more steps than the Uncertainty Penalty model (above), we however only focus on the stages concerned with the receival or anticipation of the objective reward (in our case, points). Below is the formula for how an option’s total reward value (comprised of both the discounted objective reward and anticipatory reward) is determined:

$$\begin{aligned} R\left( s_{a,}s_{o} \right)=R\left( \cdot,s_{o} \right)+\eta\tilde{R}(s_{a}|s_{o})\#\left( 3 \right) \end{aligned}$$

Where $R\left( \cdot,s_{o} \right)$ is the value of the discounted reward received in the outcome state, $\tilde{R}(s_{a}|s_{o})$ is the value of the anticipatory reward (i.e., the value derived from anticipating the delayed outcome), and $\eta$ is given by

$$\begin{aligned} \eta=\eta_{0}+c\left| E\left( s_{a},s_{o} \right) \right|\#\left( 4 \right) \end{aligned}$$

where $\eta_{0}$ is the amount of baseline anticipation and $E\left( s_{a},s_{o} \right)$ is the reward prediction error experienced when the cue is revealed (indicating whether the delayed outcome is a win or loss). We will not detail how the prediction error is determined here but we encourage the interested reader to see the original paper by Iigaya et al., (2016) or Liew et al., (2022). Returning to equation 3, the value of the discounted reward obtained at the outcome state is determined by

$$\begin{aligned} R\left( \cdot,s_{o} \right)=R_{\dagger}\left( \cdot,s_{o} \right)e^{-\gamma d}\#\left( 5 \right) \end{aligned}$$

where $R_{\dagger}\left( \cdot,s_{o} \right)$ is the objective value of the reward received in the outcome state (i.e., points) and the exponent term, $e^{-\gamma d}$, determines the extent of discounting depending on the delay length, *d*, and the free parameter, $\gamma$, which indexes an individual’s sensitivity to delay.

As previously noted, the above is a diminished description of the RPE-A model, but it gives an adequate overview of the role the objective reward plays in determining an option’s value. Analogous to the Uncertainty Penalty model, the RPE-A model specifies no direct relationship between the value of the cue state and the outcome state. While the reward received at the outcome state is devalued as a function on the delay length (equation 5), no other factors are posited to influence how much an individual values the objective reward that is received.

1. There is of course still some uncertainty as to the actual reward which will be received even it is known the eventual outcome is a ‘win’. [↑](#footnote-ref-1)
